# Picture-Word Interference Effects Are Robust With Covert Retrieval, With and Without Gamification

**DOI:** 10.3389/fpsyg.2021.825020

**Published:** 2022-01-20

**Authors:** Hsi T. Wei, You Zhi Hu, Mark Chignell, Jed A. Meltzer

**Affiliations:** ^1^Psychology Department, University of Toronto, Toronto, ON, Canada; ^2^Rotman Research Institute, Baycrest Hospital, Toronto, ON, Canada; ^3^Mechanical and Industrial Engineering Department, University of Toronto, Toronto, ON, Canada

**Keywords:** picture-word interference, word retrieval, word production, gamification, covert naming

## Abstract

The picture-word interference (PWI) paradigm has been used to investigate the time course of processes involved in word retrieval, but is challenging to implement online due to dependence on measurements of vocal reaction time. We performed a series of four experiments to examine picture-word interference and facilitation effects in a form of covert picture naming, with and without gamification. A target picture was accompanied by an audio word distractor that was either unrelated, phonologically-related, associatively-related, or categorically-related to the picture. Participants were instructed to judge whether the name of the target picture ended in the phoneme assigned to the block by pressing corresponding keys as quickly and accurately as possible. Experiments 1 and 2 successfully replicated categorical interference and phonological facilitation effects at different optimal stimulus-onset-asynchronies (SOAs) between words and pictures. Experiment 3 demonstrated that a key gamification feature (collecting coins) motivated faster speed at the expense of accuracy in the gamified vs. experimental format of the task. Experiment 4 adopted the optimal SOAs and verified that the gamification reveals expected interference and facilitation effects despite the speed-accuracy tradeoff. These studies confirmed that categorical interference occurs earlier than phonological facilitation, while both processes are independent from articulation and inherent to word retrieval itself. The covert PWI paradigm and its gamification have methodological value for neuroimaging studies in which articulatory artifacts obscure word retrieval processes, and may be developed into potential online word-finding assessments that can reveal word retrieval difficulties with greater sensitivity.

## Introduction

Object naming, a key element of everyday speech, involves rather complex word production sub-processes, such as object recognition, conceptual preparation, lexical selection, phonological encoding, and articulation (Levelt et al., 1999; Indefrey and Levelt, 2000, 2004; Levelt, 2001; Indefrey, 2011). The picture-word interference (PWI) paradigm, which presents an auditory or written distractor word with the target picture to be named, has been extensively used to investigate the time course of word retrieval processes (Glaser and Düngelhoff, 1984; Schriefers et al., 1990; Starreveld and La Heij, 1995; Roelofs et al., 1996; Alario et al., 2000; Jescheniak and Schriefers, 2001; Damian and Bowers, 2003; Costa et al., 2005; Mahon et al., 2007). Both linguistic type and stimulus onset asynchrony (SOA) of the distractor, i.e., onset of the word in relation to onset of the picture, have been found to affect naming reaction time. For example, Schriefers et al. (1990) reported that phonologically-related distractors (e.g., *fog* when the target is *dog*) sped up response if presented simultaneously (0 ms SOA) or after target picture presentation (+150 ms SOA) compared to unrelated distractors. On the other hand, categorical-semantic distractors (e.g., *cat* when the target is *dog*) slowed responses if given before picture presentation (−150 ms SOA) compared to unrelated distractors. The discrete latencies of the phonological facilitative effect and the semantic interference effect imply that the distractors interact with the targets at different stages of the word retrieval process.

Interestingly, the semantic interference effect has been specifically linked to categorical-semantic distractors (e.g., *cat* when the target is *dog*) as opposed to associative-semantic distractors (e.g., *bone* when the target is *dog*). Alario et al. (2000) found that associative-semantic distractors elicited an early facilitative effect when given before target (−234 ms SOA), and categorical-semantic distractors exerted a later inhibitory effect when given before target (−114 ms SOA). The speeding effect from associative-semantic distractors may arise from a bottom-up facilitation of conceptual activation in the earlier stages of word retrieval (La Heij et al., 2006). On the other hand, categorical-semantic distractors slowed naming responses likely because they are strengthened as competitors that meet the target's semantic/syntactic representations, blocking target name production. Regarding the different effects from semantically-related distractors, there has been a debate over the location of semantic interference in the word production process. The selection by competition account explains the semantic interference effect as the result of competition at lexical selection (Roelofs, 1992, 2001, 2003; Levelt et al., 1999; Bloem and La Heij, 2003; Damian and Bowers, 2003; Belke et al., 2005; Abdel Rahman and Melinger, 2009a,b). On the other hand, the response exclusion account explains the semantic interference as a consequence of non-lexical, articulatory motor planning that is engaged late during speaking, where the distractor word has to be removed from the response bottleneck before the target word can be articulated (Costa et al., 2005; Finkbeiner and Caramazza, 2006; Mahon et al., 2007; Janssen et al., 2008). The latter account accommodates the early semantic-associative facilitation effect by assuming that semantically-related words prime target words at the conceptual or lexical (Costa et al., 2005; Mahon et al., 2007) level, inducing facilitative, instead of inhibitory, effects. To investigate these two accounts, Abdel Rahman and Aristei (2010) compared semantic interference between PWI with and without verbal articulation in German. By observing the semantic interference effect both with and without articulation, their finding supported the selection by competition account that semantically-related distractors interfered with picture naming at the lexical selection stage, independent of the articulatory process.

Unlike semantic interference, to the best of our knowledge, phonological facilitation in PWI without verbal articulation has not yet been explored in the same manner. Phonological facilitation most likely occurs at the stage of phonological encoding before phonetic retrieval for articulation (Schriefers et al., 1990; Meyer and Schriefers, 1991; Roelofs, 1992; de Zubicaray et al., 2002; de Zubicaray and McMahon, 2009); nonetheless, its independence from articulatory planning has not been empirically tested. In this article, we develop a PWI paradigm with covert name retrieval that replicates not only the picture-word categorical interference but also the phonological facilitation effect observed in previous PWI studies with overt naming. In doing so, we aim to not only provide evidence that categorical interference and phonological facilitation are articulation-independent, supporting the theoretical word production models, but also to reveal the possibility of studying language production sub-processes without verbal responses from the participants.

Overt picture naming tasks require trained personnel to score the verbal output from participants. Manual scoring can be a time-consuming task that is vulnerable to human mistakes and low inter-rater reliability. Moreover, developing a covert naming task that investigates word production can be especially beneficial for neuroimaging studies. Functional magnetic resonance imaging (fMRI) studies have mapped the brain activity patterns that are responsible for the different sub-processes for word production (Indefrey and Levelt, 2004; see review Indefrey, 2011). Meanwhile, neurophysiological studies using magnetoencephalography (MEG) or electroencephalography (EEG) that give better temporal resolution of neural signals have provided evidence supporting the word production time course suggested by behavioral studies such as the PWI task (see review Indefrey and Levelt, 2004; Indefrey, 2011). Despite the benefit of fMRI and M/EEG for language production research, the signals collected during online speech articulation can be confounded by multiple artifactual sources (e.g., head motion, breathing, mouth movement etc.) (Yoshida et al., 1999; Gracco et al., 2005; Galgano and Froud, 2008; Chang and Glover, 2009; Tremoureux et al., 2014; Piai et al., 2015; Caballero-Gaudes and Reynolds, 2017; Power, 2017). Most neuroimaging studies on word production have sought to avoid these artifacts by either excluding the data around and at the time of verbal response, or regressing out modeled artifacts during data processing (Caballero-Gaudes and Reynolds, 2017; Abbasi et al., 2021). These methods vary greatly between studies and prevent us from understanding the neural mechanisms underlying language production in their full and unadulterated form. A demonstration that both semantic and phonological retrieval stages can be fully accessed through an experimental paradigm independent of articulatory demands would therefore open up new possibilities for experimental and clinical investigation of language production processes.

The four experiments reported in this study were approved by the Research Ethics Boards at Baycrest Hospital and the University of Toronto (#16–29). In addition to developing a PWI paradigm with covert name retrieval that induces the picture-word interference and facilitation effects, we were also interested in assessing the validity of a gamified version of our psycholinguistic paradigm. Empirical research has been moving from in-person to online administration to reduce the time and money required for data collection, especially under the social restrictions imposed during the global COVID-19 pandemic. However, remote participation entails even less control over participants' motivation and devotion, which could greatly confound experimental results through effects of boredom and fatigue (Brehman et al., 2009) arising from long and repetitive tasks, which describe most tasks in experimental psychology due to the need for adequate statistical power. The lack of participation control and the introduction of uncontrolled variance often manifest themselves as an overall slowing of reaction time in many cognitive experiments that rely on reaction time effects (Semmelmann and Weigelt, 2017). Hence, gamification can be a beneficial method to exert more control by promoting motivation and engagement for psychological experiments (Hamari et al., 2014). In this study, the gamification of the covert PWI task was facilitated by building it on the existing BrainTagger framework (see demo.braintagger.com for examples of BrainTagger games). BrainTagger is a series of whack-a-mole games that were developed initially to measure cognitive functions (Tong and Chignell, 2014; Tong et al., 2014, 2019), and for delirium screening in emergency departments (Tong et al., 2016a,b). The TAG-ME Only game for measuring response inhibition has been validated against the Go/No-Go task (Tong et al., 2019) and correlated with specific components of the Mini-Mental State Examination (Tong et al., 2020). Other than enhancing participants' engagement, gamification of the covert PWI paradigm based on the BrainTagger framework could potentially facilitate a word production assessment tool for older adults, an application that will be discussed more in depth in the section General Discussion.

## Experiment 1

The goal of Experiment 1 was to replicate established effects of slowing from categorical distractors and speeding from phonological distractors, using a covert naming design with no actual vocal production. Abel et al. (2009) reviewed the SOAs used by previous studies for different types of distractors. They managed to investigate all four linguistic distractor types—unrelated words, associatively-related words (including situational and part/whole relationships), categorically-related words, and phonologically-related words, using a single SOA (−200 ms) that elicited all the expected distractor effects in healthy young adults. They successfully replicated the speeding effect from the phonological and associative-semantic distractors and the slowing effect from the categorical distractors. Adopting Abel et al. (2009)'s method, we included four distractor types (i.e., phonological, associative, categorical, and unrelated) and used a −200 ms SOA (audio word preceding picture onset by 200 ms) between the audio distractors and picture targets.

### Materials and Methods

#### Participants

Thirty-one student participants were recruited from the University of Toronto to participate in this online study. All participants had English as their native language and had normal or corrected to normal vision and hearing. Two participants were excluded due to low accuracy (extreme outliers among all participants). Data from 29 young participants (mean age: 19.28, s.d., 3.12, age range: 18–34; 18 females) was used in the data analysis.

#### Materials

We used the SUBTLEX-US word bank to select the words for picture stimuli, filtering the wordlist to only select nouns with above 0.2 word-frequency per million words (SUBTL_WF_ ≥ 0.2) (Brysbaert and New, 2009; Brysbaert et al., 2012). The pronunciations of selected words were transcribed, using the Text to Speech Application Programming Interface (API) developed by International Business Machines Corporation (IBM), into the International Phonetic Alphabet (IPA) format with the synthesized voice “en-US_EmilyV3Voice” (https://cloud.ibm.com/apidocs/text-to-speech). The syllable counts were derived from the IPA transcription. Only words with 4 or fewer syllables were selected for the next step. To search for imageable words as picture candidates, we combined the concreteness ratings from Brysbaert et al. (2014) with the processed word-list from SUBTLEX and selected only the words with concreteness ratings of 4.5 or higher. Then, the candidate words were separated into different lists based on the ending phoneme of the word. The seven most common ending consonants (i.e., /d/, /k/, /l/, /s/, /n/, /r/, /t/) were selected as the target ending sounds in the picture-word interference paradigm. Pictures for imageable target words (words ending in target phonemes) and non-target words (words not ending in target phonemes) were selected through the Google image search engine. All pictures were screened by 30 native English-speaking North Americans recruited through Prolific (www.prolific.co), who were asked to type the first word that came to mind for each picture. Only pictures with higher than 90% name agreement were included in the study.

The pictures were separated into two sets of 144 images. List A had /d/, /k/, /l/, /n/, /r/, /t/, and list B had /k/, /l/, /s/, /n/, /r/, /t/, as the six target ending phonemes. Both lists contained 72 pictures of target words (12 pictures for each target ending phoneme) and 72 pictures of non-target words. The 144 pictures in each list were separated into 6 blocks according to the six target phonemes, each block included 12 target pictures and 12 non-target pictures. Each picture was paired with a unique audio distractor word, and was therefore limited to one experimental condition. A fully balanced design (e.g., Latin Squares) was not used because many pictures did not have suitable distractor words available for multiple conditions (e.g., phonological, categorical, associative) that met our criteria of high frequency and low syllable count. Within the 12 target and 12 non-target pictures, three pictures were paired with phonological distractors, three with associative distractors, three with categorical distractors, and three with unrelated distractors. Each picture-distractor pair only occurred once across lists A and B to avoid repetition effects. The distractors did not begin or end with the target ending phoneme of the block to avoid priming effects. Lists A and B were counterbalanced between the experimental and gamified versions of the paradigm across participants (half of the participants got List A for the experiment and List B for the game; and the other half of the participants had the reverse assignment). Each list was pre-randomized into four different orders of item presentation, and these were also counterbalanced across participants. See section 1.1 in Supplementary Material for the lists of pictures and words.

#### Conditions

Phonological distractors shared at least the beginning phoneme with the picture names (**P**; e.g., distractor: *elbow*, target: *elf* ). Associative distractors had an associative-semantic relation with the pictures (**A**; e.g., distractor: *bird*, target: *nest*). Categorical distractors were from the same semantic category as the pictures (**C**; e.g., distractor: *wasp*, target: *moth*). Unrelated distractors were words that had no apparent relation with the pictures (**UN**; e.g., distractor: *phone*, target: *shirt*). Associative and categorical distractors were selected through WordNet (Christiane, 1998) and the Word-Associations Network (project developed by Yuriy A. Rotmistrov: https://wordassociations.net/en/). We verified that associative and categorical pairs had higher semantic similarity than phonological and unrelated pairs using the pre-trained model “word2vec-google-news-300” in the “genism” package in Python. The similarity ratings for each distractor type did not pass the normality test; therefore, non-parametric one-way ANOVA (Kruskal-Wallis test) was used to compare the similarity ratings between distractors and picture names across the four conditions (*H* = 188.14, *p* < 0.001). *Post-hoc* tests utilized the Bonferroni–Holm correction to ensure that family-wise alpha was not >0.05. C and A picture-word pairs had significantly higher similarity than P and UN pairs (*p* < 0.001). Meanwhile, C pairs had significantly higher similarity than A pairs (*p* < 0.001). Then, the Kruskal-Wallis test was utilized to compare picture names between distractor conditions. The syllable counts of the picture names did not differ between distractor conditions in both list A [*H*_(3)_ = 1.87, *p* = 0.6] and list B [*H*_(3)_ = 0.76, *p* = 0.86]. The word frequency of the picture names did not differ across conditions in both list A (*H* = 1.06, *p* = 0.79) and list B (*H* = 0.42, *p* = 0.94). All distractor words were converted to audio clips using the Text to Speech API developed by IBM with the voice “en-US_LisaV3Voice” (https://cloud.ibm.com/apidocs/text-to-speech). The speech durations ranged between 720 and 1,300 ms (mean 937 ms) and did not differ significantly between conditions [*H*_(3)_ = 3.5, *p* = 0.32].

All data and the code behind this analysis have been made publicly available in the OSF repository named “Data, materials, and code for: Picture-word interference effects are robust with covert retrieval, with and without gamification” and can be accessed at doi: 10.17605/OSF.IO/K6NDP.

#### Procedure

The study was online and asynchronous. Upon signing up, participants received a google form that presented the step-by-step procedure for their participation. Participants were required to use Chrome as their web-browser to standardize the game experience, a set of headphones or earphones to help standardize audio presentation, and a keyboard for the key-pressing responses. Once participants met the requirements and consented to participate, they watched an instruction video on how to participate in the experiment and the game. They also calibrated their headphone/earphones volume with a volume calibration exercise (Woods et al., 2017) before starting the experiment.

**Figure 1:**
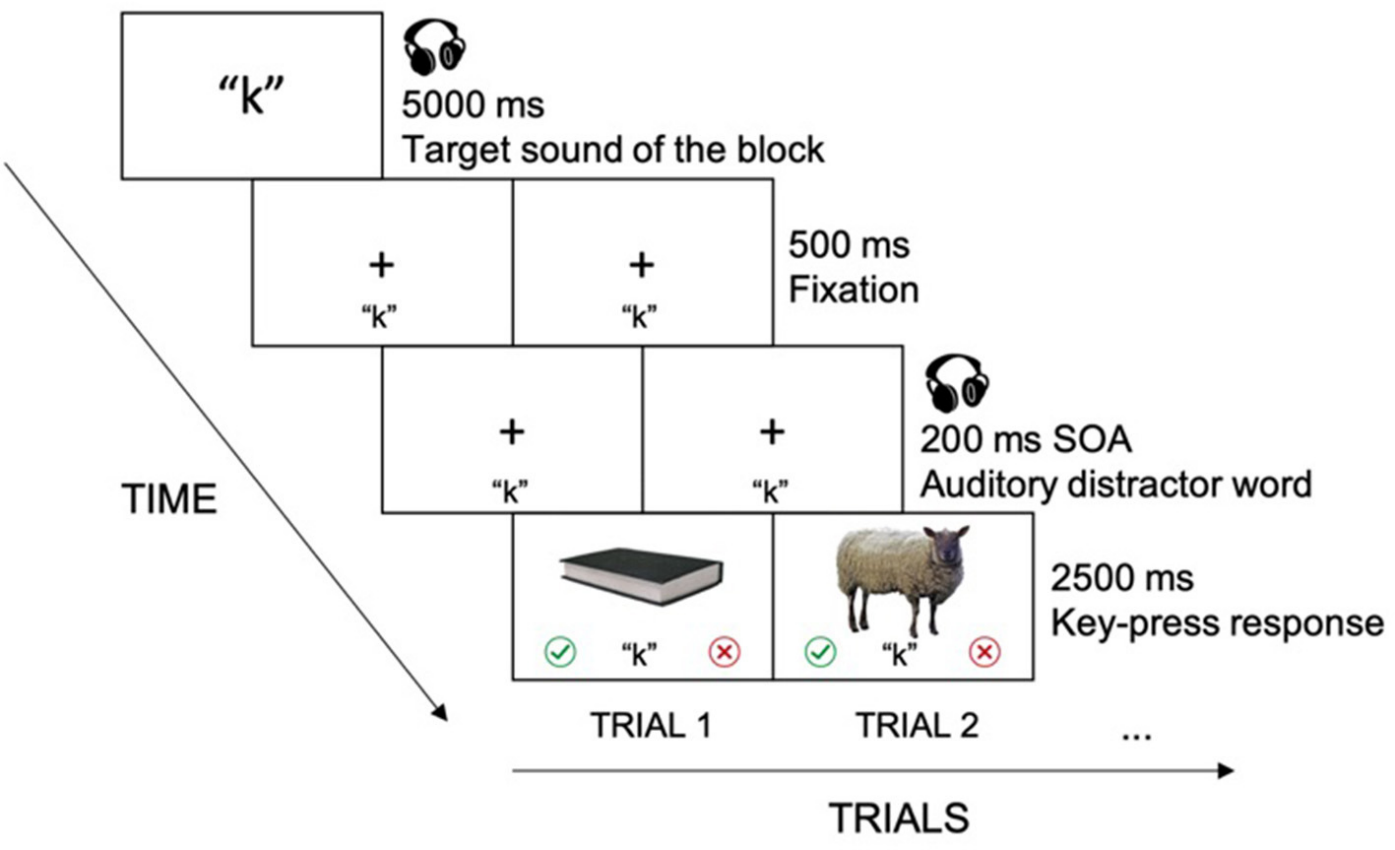
Picture-word interference experiment flow. Prior to each block, a target sound (e.g., /k/) was assigned, with a corresponding letter being presented visually and the corresponding sound being presented auditorily. In each trial, after a 500 ms fixation period, an auditory distractor word was shown with an onset of 200 ms before the onset of the target picture. The target sound of the block stayed in the lower middle of the screen so that participants would not forget the target sound. The target picture remained on the screen for 2,500 ms, and only responses (made by pressing the “J” or “K” key) during that period of time were recorded.

Participants went through two versions of the picture-word interference paradigm—the experimental version built in Psychopy (Peirce et al., 2019) and hosted by Pavlovia (https://pavlovia.org/) and the gamified version programmed and hosted by the Interactive Media Lab (Tong and Chignell, 2014; Tong et al., 2014, 2019) at the University of Toronto. The order of the two versions was counterbalanced between participants. In each version of the paradigm, participants started with a 16-trial practice session to get used to the task before they completed six blocks of the experimental tasks. At the beginning of each block, a target sound was assigned. For example, participants would see the letter for the target sound “s” and hear the sound /s/, meaning that the upcoming block had the target sound /s/. Each block was composed of 24 trials of picture name judgements. Each trial started with a 500 ms fixation. Then, a picture of a common object was presented in the middle of the screen for 2,500 ms, and an audio word was played 200 ms before the picture onset (SOA = −200). Participants were instructed to ignore the audio words (distractors) and focus on naming the object in the picture to judge whether the name of the pictured object ended in the target sound assigned for the block as quickly and accurately as possible. The judgements (answering the question of whether or not the ending phoneme of the name of the pictured object was the same as the target phoneme for the block) were made by pressing the “J” or “K” keys on the keyboard, representing the “Yes” and “No” responses, using the right hand index and middle fingers to do so. Half of the participants were assigned the “J” key as “Yes” and the “K” key as “No”; and the other half of the participants were assigned the opposite mapping. To ensure that participants had their volume on throughout the experiment, catch trials with audio instructions (presented with visual fixation) for them to press a certain key on the keyboard were inserted in each block of the experiment. This allowed us to check that the volume was on, since participants would only be able to respond correctly to the catch trials if they had the audio on to hear the instruction. See Figure 1 for the experimental flow.

**Figure 2:**
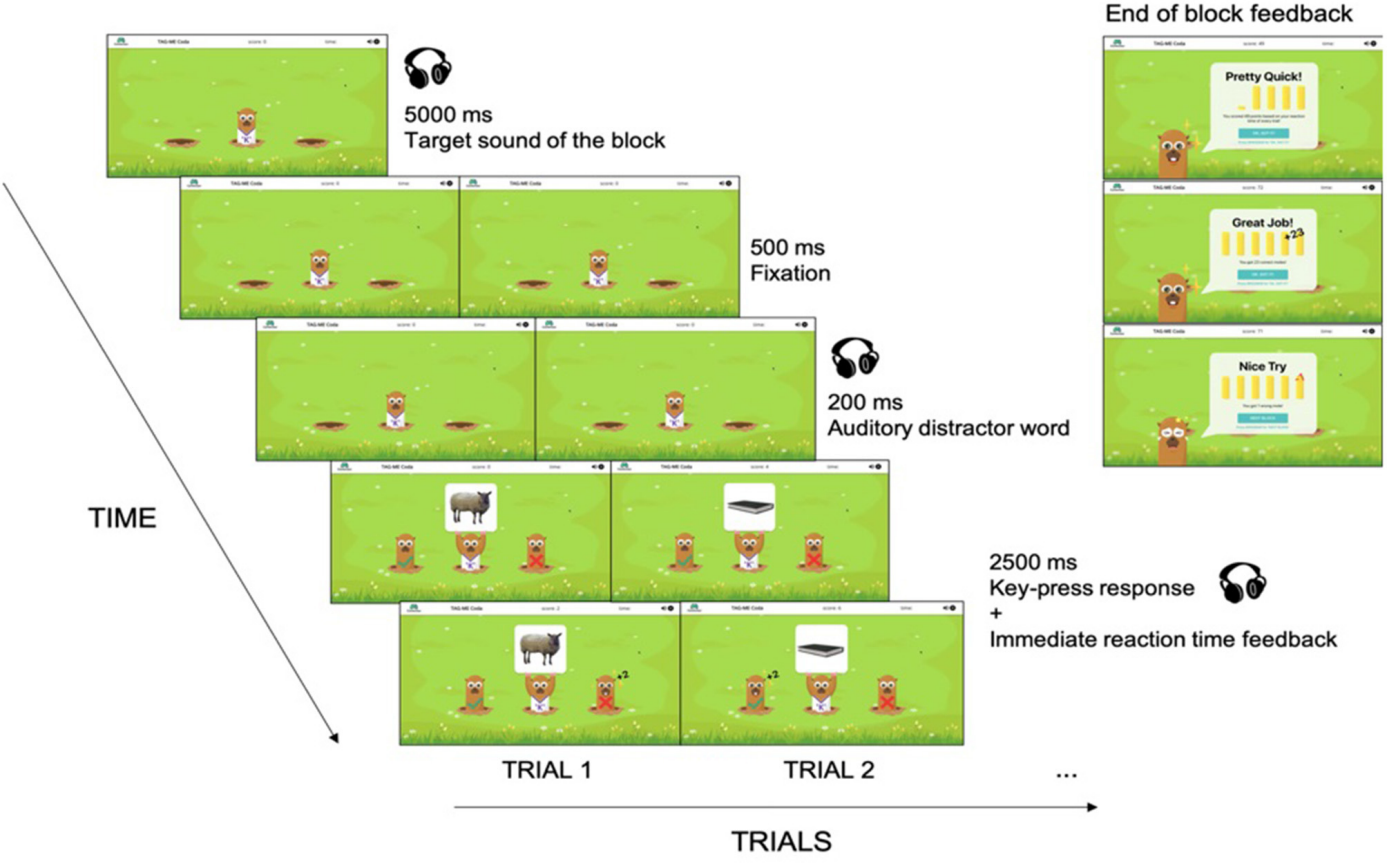
TAG-ME Coda game design flow. The same design flow from the experiment was adapted into the game. A block began with the target sound shown visually and auditorily. Each trial started with a 500 ms fixation on the middle mole, followed by an auditory distractor word played 200 ms earlier than the picture onset. Reactions were made on whether the picture name ends in the target sound of the block by pressing the “J” or “K” key. Immediate feedback was given to each response by adding points according to how fast the participant responded, accompanied by a “ding” response sound confirming the participant's button-press. Note that the game logic and timing was implemented to match the experimental task as closely as possible, but that the gamification features (i.e., animated cartoon figures, and coins that were earned based on performance) were added with the intention of making the task more enjoyable, and possibly motivating better performance.

The same picture-word interference design was utilized in the gamified version (see Figure 2). We named the game “TAG-ME Coda” since “coda” is a linguistic term referring to the ending consonant(s) of a syllable, which corresponded to the ending sound of the picture names that participants made judgements on. The same instructions (concerning the experimental task and what keys to press) were given to the participants prior to playing the game. At the beginning of each block participants saw a mole wearing a T-shirt (with the target sound of the block shown on it) while hearing the target sound twice. Once the target sound for the block was indicated in this way, data collection began for the block. On each trial, the mole in the middle pulled up a picture while two other moles popped up on both sides as the “Yes” and “No” moles. The “Yes” and “No” moles did not switch sides throughout the game and they corresponded to the “J” and “K” keys on the keyboard. The meaning of the two response keys was consistent with the experimental version. If a participant had the “J” key as “Yes” and the “K” key as “No” in the experiment, the same applied to the game, and vice versa. Audio distractors were played 200 ms before the onset of the picture. Participants were asked to judge whether the picture name ended in the target sound of the block as quickly and accurately as possible. Immediately after each response, points (visualized as coins) were added to participant total scores based on their reaction speed (the faster they responded, the more coins they earned). We decided to show immediate feedback only for reaction time but not accuracy due to our concern that participants might be unduly distracted by errors they made, affecting their performance on the subsequent trial. At the end of a block the number of total correct trials were added as bonus points while the number of incorrect trials were deducted as a penalty. The calculation of the total points they earned was shown to participants at the end of each block.

### Data Analysis

For both the experimental and gamified versions, the accuracy and correct reaction time (RT) (correct trials only) of the initial response for each trial were the primary performance measures. Two pictures in the experiment, and three pictures in the game, that yielded lower than 50% accuracy across participants were excluded from the analysis. Across all participants, 72 trials (1.7%) from the experiment and 113 trials (2.7%) from the game that had shorter than 500 ms RT were excluded since results of previous studies assessing the time course of picture naming (Levelt et al., 1998) suggested that these very quick responses were likely impulsive early responses where there was insufficient processing of the stimulus prior to making the response. The experimental and game data were grouped by distractor types. Speeding or slowing effects were examined by comparing A, C, and P distractor types to the UN distractor type, which was the baseline condition.

Since accuracy under each distractor type was not normally distributed in both experimental task and game versions (Shapiro-Wilk normality test: *p* < 0.05), the Friedman test was used to compare accuracy between distractor conditions. The Wilcoxon Signed-Ranks Test was administered as a *post-hoc* pairwise comparison, with Bonferroni–Holm correction. In contrast, RT of each condition was normally distributed for both experimental task and game versions (Shapiro-Wilk normality test: *p* > 0.05). Thus, repeated measures one-way ANOVA was used to compare RT between distractor conditions. When we observed a significant main effect, a paired *t*-test (2-tailed) was performed and thresholded for significance, adjusted by Bonferroni–Holm corrections. Pairwise comparison effect size (Cohen's *d*) was calculated by dividing the mean of differences between pairs of interest (i.e., C-UN, P-UN, A-UN) by the standard deviation of the baseline condition (UN) across participants (Cumming, 2014). Furthermore, to compare between the experimental and game versions, the Wilcoxon Signed-Ranks paired test was used to compare overall accuracy between versions while a paired *t*-test was used to compare overall RT between versions. To compare distractor effects between versions using *t*-tests, slowing and speeding effects were measured as the mean RT difference between C and UN and between UN and P, respectively, for each participant.

### Results

#### Accuracy

In the experimental version, the Friedman test revealed significant distractor effects on accuracy [$\chi_{\left(3\right)}^{2}$ = 15.01, *p* = 0.002]. Accuracy of unrelated distractors was significantly higher than that of associative distractors (*adjusted p* = 0.02) and categorical distractors (*adjusted p* = 0.001). On the other hand, no significant difference between distractor conditions were found for accuracy in the game version [$\chi_{\left(3\right)}^{2}$ = 1.48, *p* = 0.69] (see Table 1).

**Table 1:** Experiment 1: Accuracy (%) and RT (ms) are shown as mean (s.d.).

	**Cond**	**Accuracy (%)**	**Accuracy.** **p.adj.signif**	**Cohen's *d***	**RT (ms)**	**RT.** **p.adj.signif**	**Cohen's *d***
EXP version	UN	93.16 (4.7)	(baseline)		1315.61 (190.8)	(baseline)	
	C	88.4 (7.1)	**	−1.01	1364.75 (191)	*	0.26
	P	91.38 (4.8)	ns	−0.38	1325.85 (193.7)	ns	0.05
	A	89.72 (7.3)	*	−0.73	1373.18 (198.6)	**	0.3
GAME version	UN	89.61 (6.7)	(baseline)		1250.83 (189.9)	(baseline)	
	C	89.02 (7.2)	ns	−0.09	1312.02 (209)	**	0.32
	P	90.07 (5.1)	ns	0.07	1236.47 (167)	ns	−0.08
	A	90.31 (7.7)	ns	0.1	1280.56 (187.3)	ns	0.16

*p < 0.05, **p < 0.01.

#### Reaction Time (RT)

**Figure 3:**
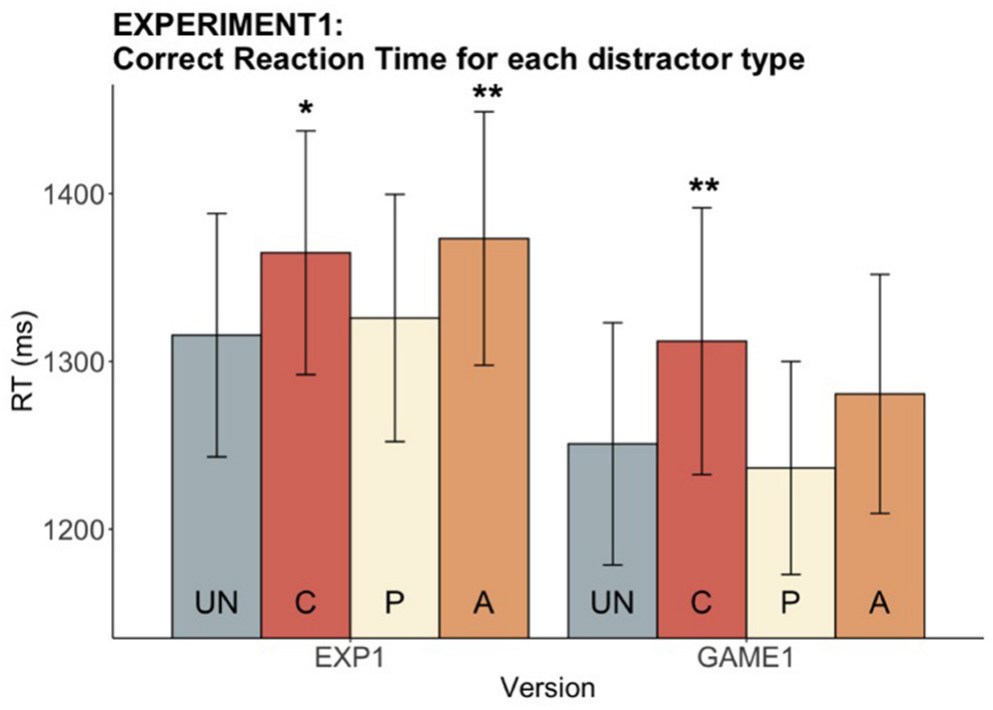
Experiment 1: Reaction time for each distractor type in both versions. The bar chart indicates the mean correct RTs while the error bars shows the 95% confidence intervals. Distractor conditions are color-coded. UN (gray; first bar) is the baseline condition that the other three conditions were compared to. Significance levels show significant difference from UN (*p < 0.05, **p < 0.01).

A significant main effect of distractor type on RT was observed in both the experimental version [*F*_(3, 84)_ = 5.63, *p* = 0.001] and the gamified version [*F*_(3, 84)_ = 9.14, *p* < 0.001] (see Figure 3). Importantly, a significant C slowing effect was found in both versions as expected [EXP: *t*_(28)_ = −2.92, *adjusted p* = 0.01; GAME: *t*_(28)_ = −3.45, *adjusted p* = 0.005]. However, we did not observe the anticipated speeding effects from P and A distractors in either paradigm version. In contrast, a slowing effect for A (compared to UN) was found in the experimental version [*t*_(28)_ = −3.49, *adjusted p* = 0.005].

#### Faster RT in Game vs. Experiment

**Figure 4:**
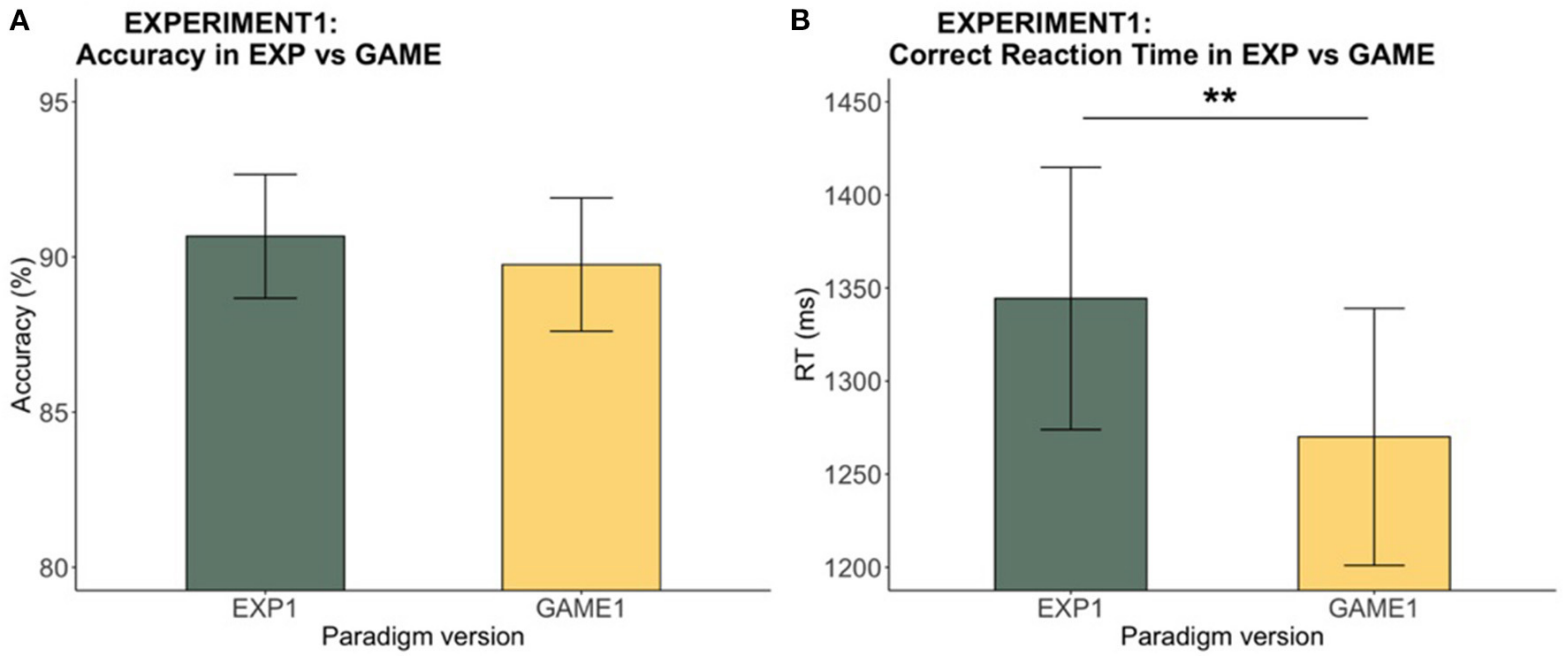
Experiment 1: Experiment vs. game accuracy and RT. The bar chart indicates **(A)** the mean accuracy and **(B)** the mean RTs across participants while the error bars shows the 95% confidence intervals. RT of the game was significantly faster than that of the experiment (**p < 0.01).

To look at the difference between the experimental and gamified versions, we compared the accuracy and RT across all participants using Wilcoxon Signed-Ranks paired tests and paired *t*-tests, respectively (see Figure 4). The response accuracy between the two versions was comparable (*V* = 292, *p* = 0.11). However, a significant difference in RT between versions was found [*t*_(28)_ = 3.29, *p* = 0.003] with RT on the game being generally faster. This result suggests that the gamified version of the paradigm had more speed motivation than the experimental version. The ceiling accuracy may have masked the potential speed accuracy tradeoff between versions. To look at whether the RT difference between the two versions affected the slowing and speeding effects, each participant's slowing effect was taken as the mean RT difference between C and UN (C-UN) and the speeding effect as the mean RT difference between UN and P. Dependent *t*-tests indicated that no significant difference was found in the slowing effect [*t*_(28)_ = −0.55, *p* = 0.59] or the speeding effect [*t*_(28)_ = −0.96, *p* = 0.35] between the two versions, despite the overall RT difference.

### Discussion

In Experiment 1, we replicated the slowing effect from categorical distractors (RT: C > UN) in both the experimental and the gamified versions. Interestingly, we observed that gamification of the task had an effect on the overall reaction speed while preserving the degree of the slowing effect (C-UN). However, we did not observe significant speeding effects from phonological distractors and associative distractors. Suboptimal selection of SOA could be a potential reason why we did not observe a speeding effect from phonological distractors. As shown by the review done by Abel et al. (2009), the phonological speeding effect has been most reliably elicited with 0 or positive SOA, instead of −200 SOA (Schriefers et al., 1990; Damian and Martin, 1999; Jescheniak and Schriefers, 2001). Thus, a positive SOA may be required for the phonological speeding effect to occur, because phonological processes occur later in word production. On the other hand, associative distractors actually slowed picture naming in the experimental version. Although an associative speeding effect was found with −200 SOA by Abel et al. (2009), −200 SOA could arguably be too late since associative speeding effect may occur at an even earlier timepoint as suggested by Alario et al. (2000). Moreover, associative facilitation has always been a weaker effect than categorical interference as observed in the literature (La Heij et al., 1990; Alario et al., 2000; Abel et al., 2009). Also, activation strength of the distractor has been suggested to impact whether a semantically-related picture-word pair would result in an interference or facilitation, where a stronger distractor activation results in interference and a weaker distractor activation (by masking the distractors) elicits facilitation (Damian and Spalek, 2014). It is possible that the activation strength of the associative distractors was too strong in this experiment, thus resulting in an interference effect. However, the interaction between activation strength of distractors and the polarity of picture-word effect is not the focus of this study. Future studies are required to further examine this account.

## Experiment 2

The goal of Experiment 2 was to test the impact of a potentially more suitable picture-word SOA for revealing the anticipated phonological speeding effect that was absent in Experiment 1. First, we adjusted the SOA from −200 to 0 ms for all distractor types. Second, we replaced the associative distractors with a new condition with no distractors. Because the associative condition was not of primary interest in the present study, and also because associative facilitation is even less likely to occur with a later SOA as used here, we decided to instead add a different comparison condition that was also of interest but could not be feasibly tested in the first experiment. Adding a condition with no distractor allowed comparison of slowing and speeding effects against picture naming when there were no distractors, and specifically allowed us to test whether phonological “speeding” would reduce reaction time below that seen with no distractor at all, or to a level intermediate between the unrelated distractor and no distractor levels.

### Materials and Method

#### Participants

Thirty-three participants (mean age: 23.21, s.d., 5.69, age range: 18–34; 19 females) were recruited from the University of Toronto, and through the Prolific (www.prolific.co) online recruitment platform, to participate in this study. All participants had English as their native language and had normal or corrected to normal vision and hearing.

#### Materials, Conditions, and Procedure

Most of the same picture and audio materials from Experiment 1 were used for Experiment 2. The two changes to the experimental procedure for Experiment 2 were that the associative condition was changed to a no distractor condition (the audio distractor words previously paired with the associative pictures were removed from Experiment 2, as can be seen from viewing the lists of stimuli in section 1.2 in Supplementary Material) and the SOA was changed from −200 to 0 ms in both the experimental and gamified versions of the paradigm.

### Data Analysis

For both the experimental and gamified versions, the accuracy and correct reaction time (RT) (correct trials only) of initial responses were analyzed. Two pictures in the experiment, and six pictures in the game, that yielded lower than 50% accuracy across participants were excluded from the analysis. Across all participants, 87 trials (1.8%) from the experiment and 86 trials (1.8%) from the game with shorter than 500 ms RT were excluded. Speeding or slowing effects were examined by comparing the P and C distractor types to the UN distractor type. The same statistical analyses from Experiment 1 were used in Experiment 2 to discover distractor effects on accuracy and RT, as well as the gamification effects. To compare distractor effects between task and game versions using *t*-tests, slowing and speeding effects were separated out by taking the mean RT difference between C and UN, and between UN and P, respectively, for each participant.

### Results

#### Accuracy

No main effect of distractor type was found on accuracy in the experimental version [$\chi_{\left(3\right)}^{2}$ = 3.46, *p* = 0.33]. A significant distractor effect was found in the gamified version of the paradigm [$\chi_{\left(3\right)}^{2}$ = 10.9, *p* = 0.01]. However, none of the pairwise comparisons yielded a significant adjusted *p*-value (see Table 2).

**Table 2:** Experiment 2: Accuracy (%) and RT (ms) are shown as mean (s.d.).

	**Cond**	**Accuracy (%)**	**Accuracy.** **p.adj.signif**	**Cohen's *d***	**RT (ms)**	**RT.** **p.adj.signif**	**Cohen's** ***d***
EXP version	UN	89.38 (9.4)	(baseline)		1315.67 (225)	(baseline)	
	C	89.72 (8.6)	ns	0.04	1332.73 (225.2)	ns	0.08
	P	91.83 (7)	ns	0.26	1260.84 (211.8)	**	−0.24
	No	90.36 (8.9)	ns	0.1	1274.43 (200.6)	**	−0.18
GAME version	UN	86.88 (10.2)	(baseline)		1175.53 (202.5)	(baseline)	
	C	84.44 (9.9)	ns	−0.24	1218.21 (206.2)	*	0.21
	P	88.06 (10.61)	ns	0.12	1140.93 (101.7)	*	−0.17
	No	85.3 (9)	ns	−0.15	1146.56 (193.3)	*	0.14

*p < 0.05, **p < 0.01.

#### Reaction Time (RT)

**Figure 5:**
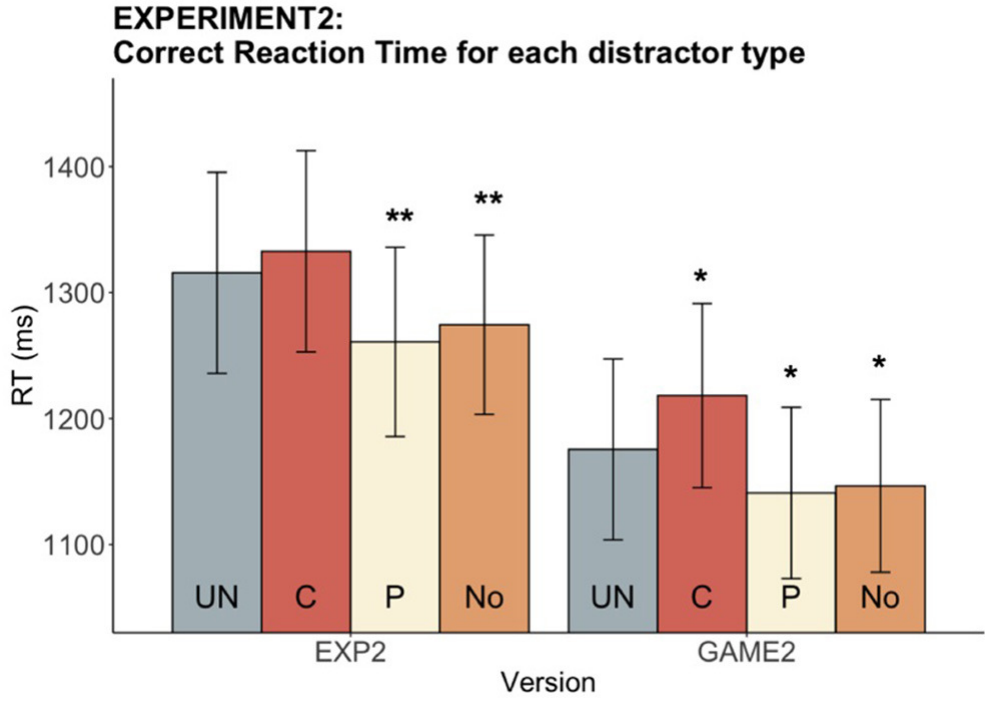
Experiment 2: Reaction time for each distractor type in both versions. The bar chart shows mean RTs while the error bars show 95% confidence intervals. The distractor conditions are color-coded, with the UN (gray) being the baseline condition for pairwise statistical comparisons with the other three conditions. Asterisks indicate that mean RT for the corresponding condition differs significantly from the UN mean RT (*p < 0.05, **p < 0.01).

A significant main effect of distractor type on RT was found in both the experimental [*F*_(3, 96)_ = 10.84, *p* < 0.001] and gamified versions [*F*_(3, 96)_ = 12.89, *p* < 0.001]. As expected, a significant P speeding effect was found in both versions [EXP: *t*_(32)_ = 3.52, *adjusted p* = 0.004; GAME: *t*_(32)_ = 2.76, *adjusted p* = 0.02]. However, a significant C slowing effect was only found in the gamified version [*t*_(32)_ = −2.88, *adjusted p* = 0.021]. In both versions, RT of No distractor was significantly faster than that of UN [EXP: *t*_(32)_ = −3.24, *adjusted p* = 0.008; GAME: *t*_(32)_ = −2.77, *adjusted p* = 0.03]. These results confirm that 0 SOA elicited a speeding effect from phonological distractors, and that due to this speeding participants named pictures as fast as when there was no distractor (see Figure 5). Moreover, no categorical slowing effect was found in the experimental version, suggesting that 0 SOA may not be as effective in inducing a slowing effect as was the −200 SOA used in Experiment 1.

#### Speed-Accuracy Tradeoff Between Experiment and Game

**Figure 6:**
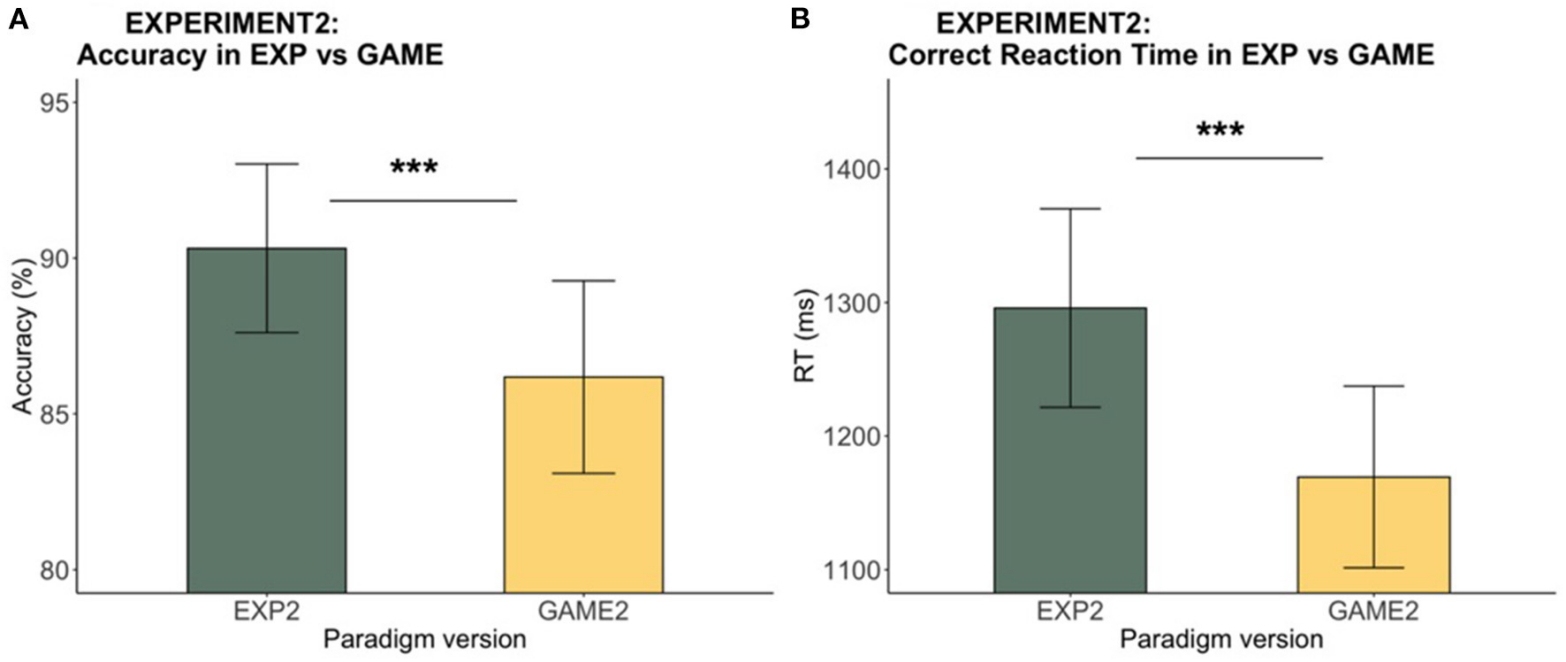
Experiment 2: Experiment vs. game accuracy and RT. Figure shows **(A)** the mean accuracy, and **(B)** the mean RTs, across participants. The error bars show 95% confidence intervals. Accuracy of the game was lower, and RT of the game was faster, as compared to the experiment. A speed-accuracy tradeoff was observed between the two versions of the paradigm (***p < 0.001).

Accuracy and RT were compared between the experimental and gamified versions to further characterize the version effects (see Figure 6). We observed a significantly higher accuracy in the experiment than in the game (V = 505, *p* < 0.001). Meanwhile, a significantly faster RT was observed in the game as compared to the experiment [*t*_(32)_ = 6.18, *p* < 0.001]. Thus, there was an overall speed-accuracy tradeoff between the two versions where participants reacted faster but less accurately in the game. To compare the speeding and slowing effects from the two versions, we took the difference between P and UN as the amount of speeding effect, and the difference between C and UN as the amount of slowing effect, and compared the speeding and slowing effects between the two versions with a paired *t*-test. No significant difference in the speeding and slowing effects between the experiment and the game was found at the participant level [UN-P speeding: *t*_(32)_ = 1.05, *p* = 0.30; C-UN slowing: *t*_(32)_ = −1.27, *p* = 0.21].

#### −200 SOA vs. 0 SOA for Distractor Effects

In Experiment 1, with −200 SOA, we observed a consistent C slowing effect in both versions of the paradigm, but no P speeding effect was detected. On the other hand, in Experiment 2 with 0 SOA, we found a consistent P speeding effect across both versions while the slowing effect was only obtained in the gamified version. To select the ideal picture-word SOAs for the slowing and speeding effects, we took the difference between C and UN as the slowing effect and the difference between UN and P as the speeding effect for both Experiment 1 and Experiment 2. Independent *t*-tests were utilized to compare the slowing and speeding effects between Experiment 1 and 2. For the P speeding effect, we found a significantly stronger speeding effect in Experiment 2 with 0 SOA compared to Experiment 1 with −200 SOA [*t*_(60)_ = 2.72, *p* = 0.009]. No significant speeding effect difference between game 1 with 0 SOA and game 2 with −200 SOA was found [*t*_(60)_ = 1.03, *p* = 0.31]. The result suggests that the 0 SOA has a better sensitivity in revealing the phonological speeding effect than the −200 SOA in the experimental version of the paradigm. On the other hand, for the C slowing effect, no significant difference was found between Experiment 1 and Experiment 2 for the experimental task version [*t*_(60)_ = −1.43, *p* = 0.16] nor between Experiments 1 and 2 for the game version [*t*_(60)_ = −0.81, *p* = 0.42].

### Discussion

Combining the results from Experiments 1 and 2, we found that an SOA of 0 ms is better at eliciting a phonological speeding effect than an SOA of −200 ms. The phonological speeding effect may be more prominent when there is no SOA because phonological processes happen at a later stage of the picture naming process (Mackay, 1988; Levelt et al., 1999) as supported by previous picture-word interference studies (Schriefers et al., 1990; Damian and Martin, 1999). On the other hand, significant slowing due to the category effect (C compared to UN) was only observed for the experimental task in Experiment 1 (and not in Experiment 2), suggesting that an SOA of 0 ms might not be reliable in producing the categorical slowing effect. Moreover, categorical distractors were expected to have a stronger effect with an earlier SOA (Schriefers et al., 1990; Damian and Martin, 1999; Alario et al., 2000) since categorical distractors interfere with the picture naming process at earlier stages of sensory recognition or lexical selection (Levelt et al., 1999; Abel et al., 2009). Based on the theoretical processes underlying picture naming (Mackay, 1988; Levelt et al., 1999), previous literature on picture-word interference (Schriefers et al., 1990; Damian and Martin, 1999; Alario et al., 2000; Abel et al., 2009), and our current findings in Experiment 1 and 2, we identified an SOA of −200 ms for categorical distractors, and 0 ms for phonological distractors, as the optimal SOA settings for our picture-word interference paradigm. We decided to use one unrelated distractor condition with an SOA of −200 ms, and another unrelated distractor condition with an SOA of 0 ms, in the subsequent experiments reported below. This allowed us to have baseline (comparison) conditions with SOAs that matched each of the two conditions. Before we tested the final paradigm with the selected SOA parameters (see Experiment 4), we carried out Experiment 3 to further investigate the speed-accuracy trade-off observed between the experiment and the game in Experiment 2. We hypothesized that the speed-accuracy tradeoff between versions was driven by the gamification incentives for the participants to respond fast (e.g., the faster one responded, the more coins one earned). To test this hypothesis, we recruited a new group of participants in Experiment 3, in which the experimental design remained the same as in Experiment 2, but all the speed incentives in the game were removed.

## Experiment 3

The goal of Experiment 3 was to test whether the coin-rewarding design of the game was the driving factor behind the speed-accuracy tradeoff observed in Experiment 2. Experiment 3 utilized the same experimental design as Experiment 2. An SOA of 0 ms was used between word and picture for each trial with no distractors, unrelated distractors, categorical distractors, and phonological distractors as the four different distractor types for both versions of the paradigm. However, in the game, we removed the immediate feedback for coins earned by RT for each trial along with the animation for coins calculation at the end of each block.

### Materials and Methods

#### Participants

Thirty-five participants were recruited from University of Toronto and through Prolific (www.prolific.co), to participate in this online study. One participant was excluded for failing to respond to the audio catch trials in the experiment. Another participant was excluded for low accuracy (extreme outliers among all participants). Data for the remaining 33 participants (mean age: 22.85, s.d., 5.19, age range: 18–34; 18 females) was used in the data analysis. All participants had English as their native language and had normal or corrected to normal vision and hearing.

#### Materials, Conditions, and Procedure

All procedures were identical to Experiment 2 except for the removal of the coin incentive elements from the gamified version.

### Data Analysis

For both the experimental and gamified versions, the accuracy and correct reaction time (RT) (correct trials only) of initial responses were analyzed. Data from three pictures in the experiment, and five pictures in the game, that yielded lower than 50% accuracy across participants were excluded from the analysis. Across all participants, 62 trials (1.3%) from the experiment and 310 trials (5.9%) from the game with shorter than 500 ms RT were also excluded. The same statistical analyses used in Experiments 1 and 2 were again used in Experiment 3 to evaluate distractor effects on accuracy and RT, as well as the gamification effects.

### Results

#### Accuracy

No main effect of distractor type on accuracy was found in the experimental version of the paradigm [$\chi_{\left(3\right)}^{2}$ = 3.6, *p* = 0.3]. A significant distractor effect on accuracy was observed in the gamified version [$\chi_{\left(3\right)}^{2}$ = 9.34, *p* = 0.02]. However, no pairwise comparison yielded a significant adjusted *p*-value (see Table 3).

**Table 3:** Experiment 3: Accuracy (%) and RT (ms) are shown as mean (s.d.).

	**Cond**	**Accuracy (%)**	**Accuracy.** **p.adj.signif**	**Cohen's** ***d***	**RT (ms)**	**RT.** **p.adj.signif**	**Cohen's** ***d***
EXP	UN	88.64 (9.5)	(baseline)		1302.8 (155)	(baseline)	
version	C	89.09 (8.6)	ns	0.05	1353.07 (166.4)	**	0.32
	P	91.4 (6.1)	ns	0.29	1255.77 (152.9)	*	−0.3
	No	87.94 (8.2)	ns	−0.07	1258.15 (158.1)	*	−0.29
GAME	UN	88.76 (8)	(baseline)		1315.84 (175.1)	(baseline)	
version	C	87.38 (8.1)	ns	−0.17	1366.47 (174.1)	**	0.29
	P	90.08 (7.3)	ns	0.17	1260.01 (147)	**	−0.32
	No	90.53 (6.3)	ns	0.22	1293.14 (166)	ns	−0.13

*p < 0.05, **p < 0.01.

#### Reaction Time (RT)

**Figure 7:**
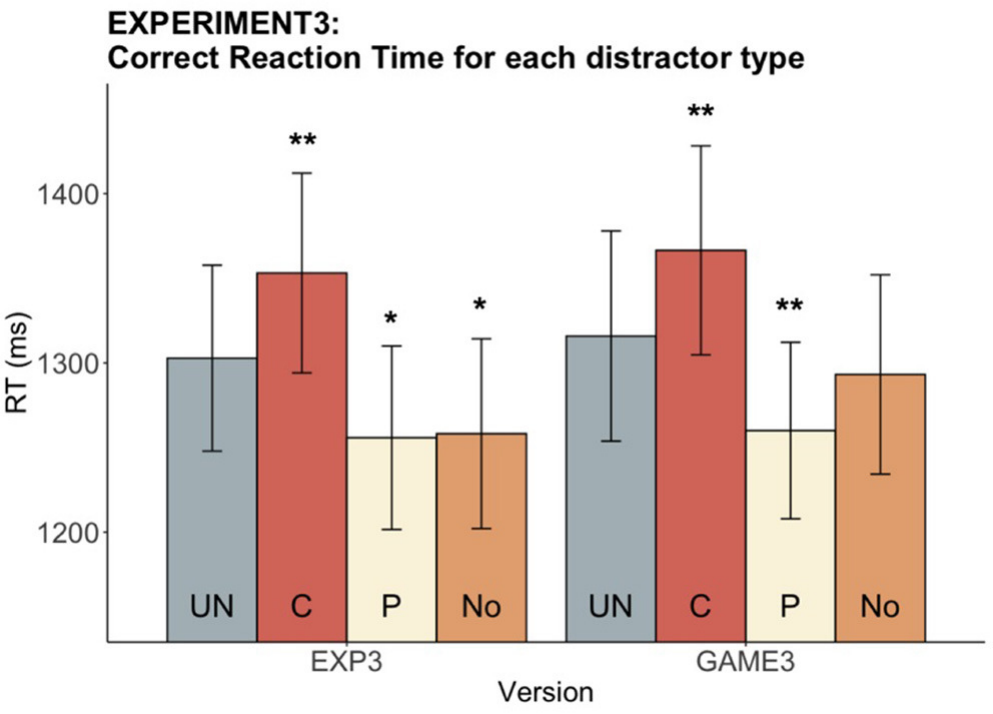
Experiment 3: Reaction time for each distractor type in both versions. The bar chart indicates the mean RTs while the error bars shows the 95% confidence intervals. The distractor conditions are color-coded, with the UN (gray) being the baseline condition for pairwise statistical comparisons with the other three conditions. Asterisks indicate that mean RT for the corresponding condition differs significantly from the UN mean RT (*p < 0.05, **p < 0.01).

A main distractor effect on RT was found in both the experiment [*F*_(3, 96)_ = 19.7, *p* < 0.001] and the game [*F*_(3, 96)_ = 17.11, *p* < 0.001]. Significant C slowing and P speeding effects were observed in both versions [EXP speeding: *t*_(32)_ = 2.92, *adjusted p* = 0.01, slowing: *t*_(32)_ = −3.74, *adjusted p* = 0.002; GAME: speeding: *t*_(32)_ = 3.46, *p* = 0.003, slowing: *t*_(32)_ = −3.58, *p* = 0.003]. RT of the No-distractor condition was significantly faster than UN in the experiment [*t*_(32)_ = 2.52, *adjusted p* = 0.02] but not in the game [*t*_(32)_ = 1.48, *adjusted p* = 0.15]. We successfully elicited the P speeding and C slowing effects in both versions, finding that P speeding facilitated naming to the same extent as, or even faster than, naming without distractor in our paradigm (see Figure 7).

#### Speed-Accuracy Tradeoff Removed

**Figure 8:**
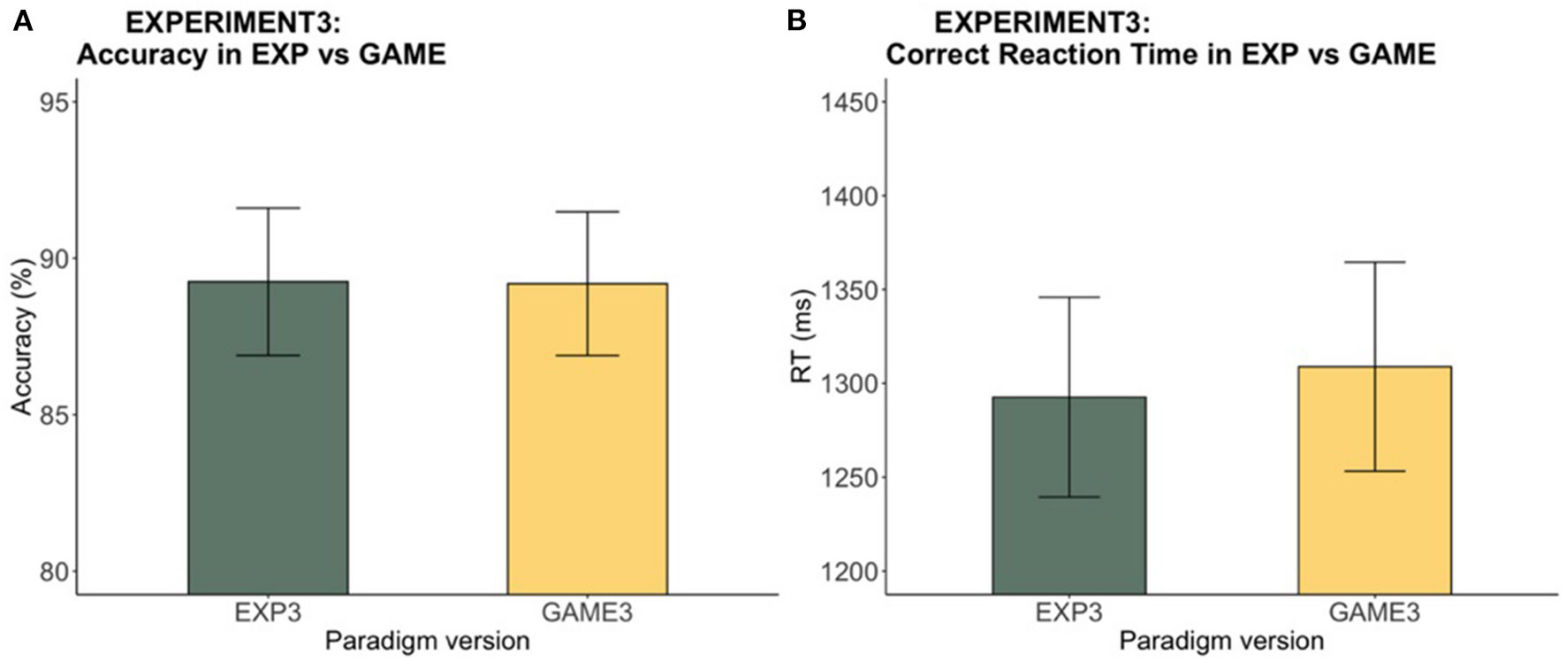
Experiment 3: Experiment vs. game accuracy and RT. The bar chart indicates **(A)** the mean accuracy and **(B)** the mean RTs across participants, while the error bars show the 95% confidence intervals. No significant difference was found in accuracy or RT between versions.

Accuracy and RT were compared between the experimental and gamified versions in Experiment 3 to assess possible version effects. No significant difference between the two versions was found in either accuracy (*V* = 287, *p* = 0.92) or RT [*t*_(32)_ = −0.87, *p* = 0.39]. Unlike what we saw from Experiment 2, there was no speed-accuracy tradeoff between the experiment and the game in Experiment 3 (see Figure 8), supporting our hypothesis that the speed-motivation elements in the game were the reasons behind the speed-accuracy trade of between experiment and game. Once we removed the design elements inducing speed motivation in the game, we removed the tendency for increased speed and decreased accuracy in the game.

#### Game With vs. Without, Speed Motivation

**Figure 9:**
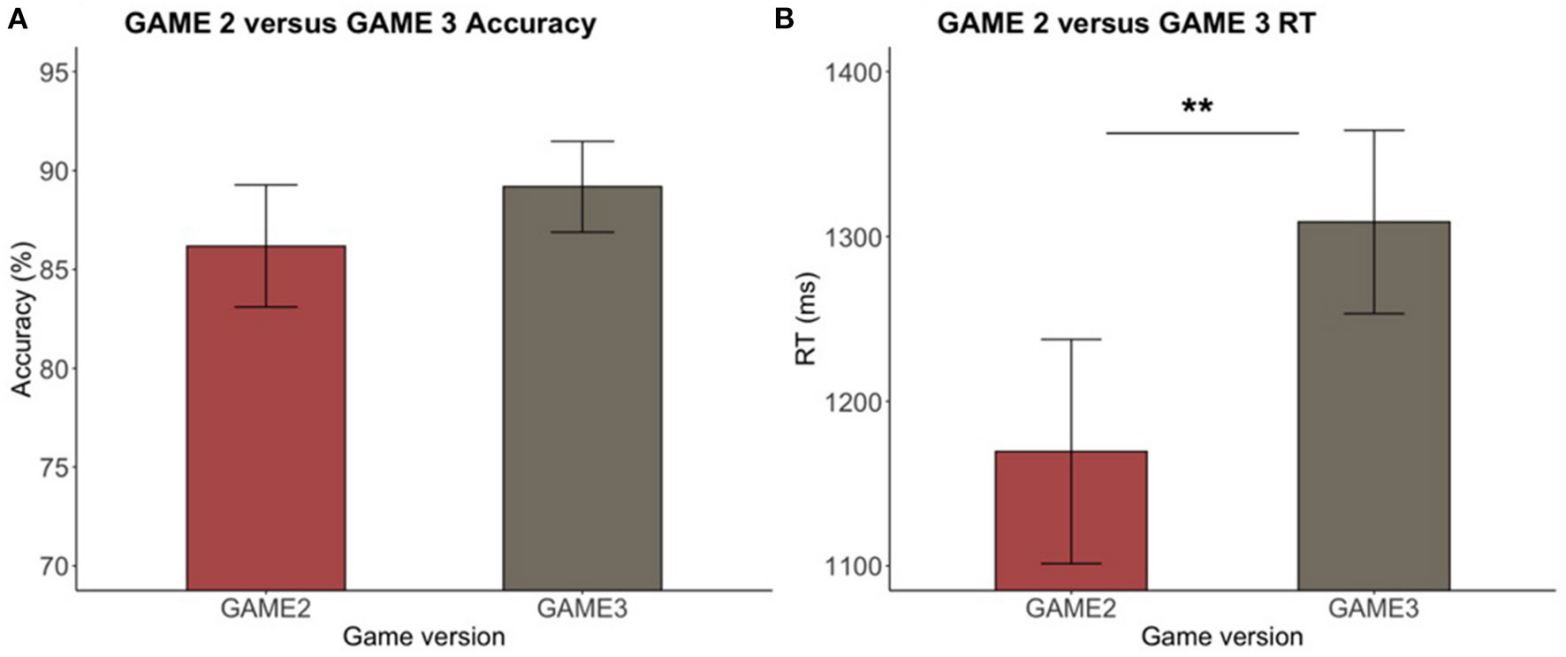
**(A,B)** Game version 2 vs. game version 3 accuracy and RT. No significant difference was found in accuracy while a significant difference was found in RT between versions (**p < 0.01; error bar = 95% CI).

A two-sample independent *t*-test was used to investigate accuracy and RT differences between the game version with speed motivation (game v2) and without speed motivation (game v3). The RT of game version 2 was significantly faster than that of game version 3 [*t*_(64)_ = −3.21, *p* < 0.01] (see Figure 9). Nonetheless, no significant difference was found between the accuracy of the two versions [*t*_(64)_ = 0.16, *p* = 0.87]. The P speeding (UN – P), and C slowing (C – UN), effects were compared between game versions using another set of independent *t*-tests. No significant differences were found in the magnitude of the speeding [*t*_(64)_ = −1.04, *p* = 0.3] or slowing [*t*_(64)_ = −0.39, *p* = 0.7] effects between the two versions of the game despite the overall faster RT in the game version that included the coin reward feature that motivated speed.

### Discussion

By removing the speed incentives in the game, the speed-accuracy tradeoff between the experiment and game disappeared, supporting our hypothesis that the game's coin-reward feature was the driver behind the speed-accuracy tradeoff between versions observed in Experiment 2. So far, our findings from the three Experiments support the validity of gamifying the picture-word interference paradigm. Despite the speed-accuracy tradeoff between the experiment and the game with speed incentives, the target slowing and speeding effects can be reliably elicited with the optimal SOAs (as discussed in Experiment 2) in both versions of the paradigm. Although we observed the slowing effect in Experiment 3 with the SOA of 0 ms, our earlier choice of selecting −200 SOA for the categorical slowing effect is supported by the relevant research literature (Schriefers et al., 1990; Damian and Martin, 1999; Levelt et al., 1999; Alario et al., 2000; Abel et al., 2009) and the absence of the slowing effect in experiment 2 with an SOA of 0 ms. Additionally, Experiment 3 provides insight into the impact of the coin-reward gamification feature. Despite the overall shorter RT in the game with speed motivation compared to the game without, no significant difference in the speeding and slowing effects, or the accuracy, was found between the two versions of the game. Based on this finding, we are confident that the fully gamified version of the game (including speed incentives) can reliably elicit the slowing and speeding effects expected while maintaining a sufficiently high accuracy rate.

## Experiment 4

Based on the findings from the previous three Experiments, we ran one more experiment to assess the optimal SOAs with 4 distractor types (UN with −200 SOA, UN with 0 SOA, C with −200 SOA, and P with 0 SOA) for both the experimental and gamified version of the picture naming paradigm, and this experiment also included the speed motivation (coin-reward) for the game. The goal of Experiment 4 was to validate this final experimental methodology in terms of its ability to reveal P speeding and C slowing effects in both the experimental and gamified versions.

### Materials and Methods

#### Participants

Forty-three participants were recruited from University of Toronto to participate in this online study. Two participants were excluded for low accuracy (extreme outliers among all participants). Forty-one participants (mean age: 19.41, s.d., 2.8, age range: 18–34; 29 females, 1 non-binary) were included in the data analysis. All participants had English as their native language and had normal or corrected to normal vision and hearing.

#### Materials, Conditions, and Procedure

Experiment 4 used the same procedure as the previous Experiments. A 0 SOA was used for P distractors and a −200 SOA was used for C distractors. In order to allow baseline comparisons with matching SOAs for P and C distractors, two UN conditions, one at 0 SOA (UN_0) and one at −200 SOA (UN_200) were included. Most of the same picture and audio materials from Experiment 2 and 3 were also used in Experiment 4. The only difference was that the No distractor condition was changed to a UN_200 condition. Consequently, a new set of unrelated distractor words were added to accommodate the additional UN condition used in this study (see section 1.3 in Supplementary Material for stimuli lists).

### Data Analysis

For both the experimental and gamified versions, the accuracy and correct reaction time (RT) (correct trials only) of initial responses were analyzed. Four pictures in the experiment, and three pictures in the game, that yielded lower than 50% accuracy across participants were excluded from the analysis. Across all participants, 104 trials (1.7%) from the experiment and 90 trials (1.5%) from the game with shorter than 500 ms RT were excluded. The same statistical analyses from the previous three Experiments were used on the Experiment 4 data to discover distractor effects on accuracy and RT, and gamification effects. P was compared with UN_0 as the baseline condition, while C was compared with UN_200 as the baseline condition.

### Results

#### Accuracy

A significant distractor effect on accuracy was observed in both the experimental [$\chi_{\left(3\right)}^{2}$ = 9.21, *p* = 0.03] and the gamified version [$\chi_{\left(3\right)}^{2}$ = 17.52, *p* < 0.001]. Focusing on the comparison pairs of interest, no significant difference was found between the accuracy of P_0 and UN_0 or between C_200 and UN_200 in the experimental version. However, in the game version, P_0 had a significantly higher accuracy than UN_0 (*adjusted p* =.02), consistent with a phonological priming effect in picture naming (see Table 4).

**Table 4:** Experiment 4: Accuracy (%) and RT (ms) are shown as mean (s.d.).

	**Cond**	**Accuracy (%)**	**Accuracy.** **p.adj.signif**	**Cohen's** ***d***	**RT (ms)**	**RT.** **p.adj.signif**	**Cohen's** ***d***
EXP	UN_200	87.4 (8)	(baseline for C_200)		1260.83 (126.4)	(baseline for C_200)	
version	C_200	87.23 (6.7)	ns	−.02	1331.23 (143.7)	****	0.56
	UN_0	88.91 (8.4)	(baseline for P_0)		1354.62 (140.6)	(baseline for P_0)	
	P_0	90.56 (6.2)	ns	0.2	1309.07 (142.6)	**	−0.32
GAME	UN_200	87.11 (7.1)	(baseline for C_200)		1213.97 (131.2)	(baseline for C_200)	
version	C_200	86.55 (7.9)	ns	−.07	1271.27 (153)	***	0.44
	UN_0	87.33 (8.1)	(baseline for P_0)		1251.13 (123.2)	(baseline for P_0)	
	P_0	90.52 (6.9)	*	0.39	1203.47 (132)	**	−0.39

*p < 0.05, **p < 0.01, ***p < 0.001, ****p < 0.0001.

#### Reaction Time (RT)

**Figure 10:**
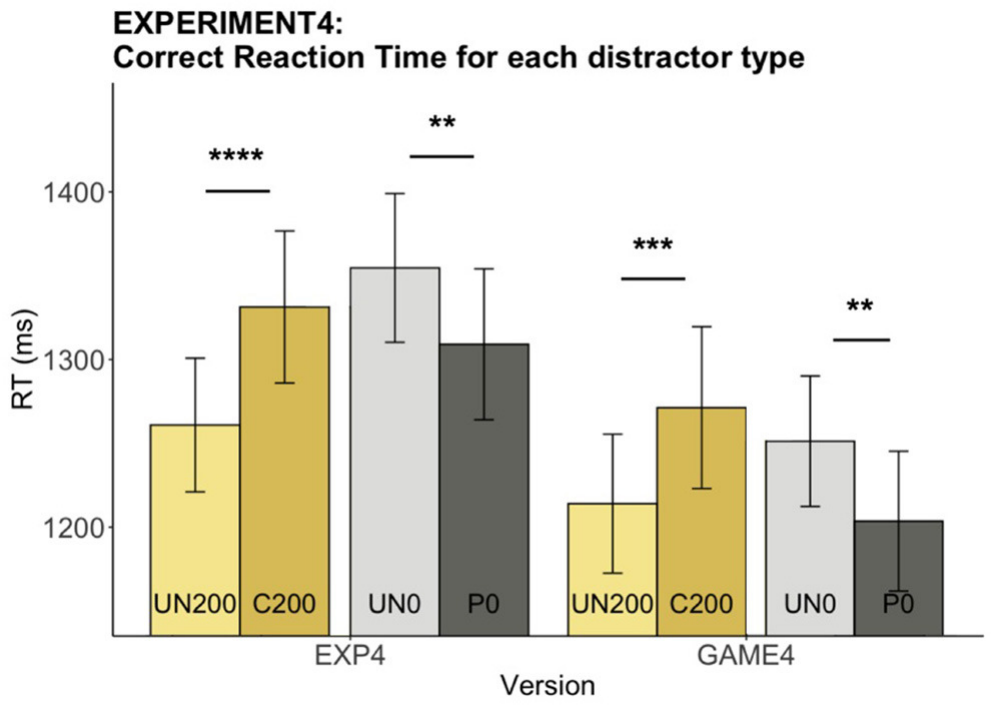
Experiment 4: Reaction time for each distractor type in both versions. The bar charts indicate mean RTs while overlaid error bars show 95% confidence intervals. The distractor conditions are color-coded. UN_200 is the baseline condition for C_200 and UN_0 is the baseline condition for P_0. Asterisks indicate that mean RT for the corresponding condition differs significantly from its baseline condition mean RT (**p < 0.01. ***p < 0.001, ****p < 0.0001).

A main distractor effect on RT was found in both the experiment [*F*_(3, 120)_ = 15.96, *p* < 0.0001] and the game [*F*_(3, 120)_ = 10.28, *p* < 0.0001] (see Figure 10). As expected, significant C slowing and P speeding effects were observed in both versions [EXP slowing: *t*_(40)_ = −5.22, *adjusted p* < 0.001, speeding: *t*_(40)_ = 3.25, *adjusted p* < 0.01; GAME: slowing: *t*_(40)_ = −4.29, *p* < 0.001, speeding: *t*_(40)_ = 3.95, *p* = 0.001]. The results demonstrate that the designs of Experiment 4 can elicit the speeding and slowing effects of interest.

#### Faster RT in Game vs. Experiment

**Figure 11:**
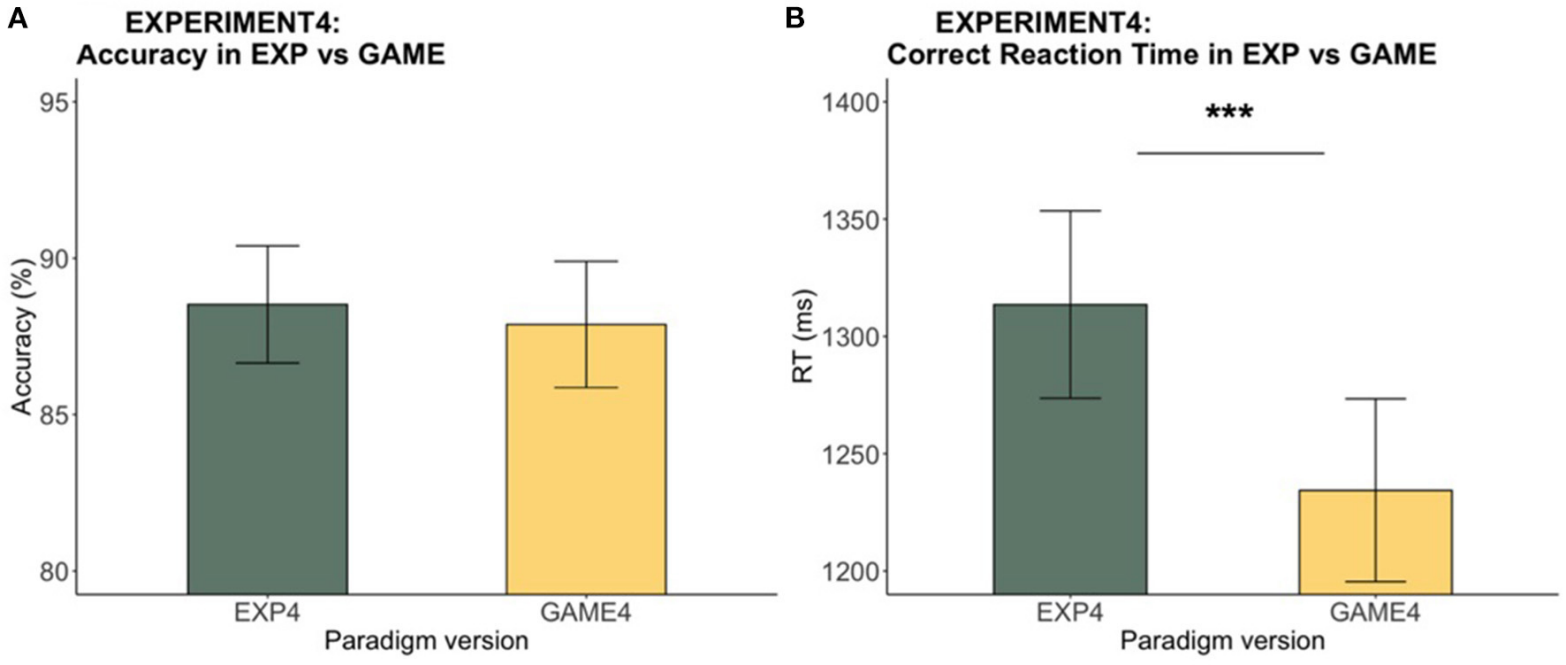
Experiment 4: Experiment vs. game accuracy and RT. The bar chart indicates **(A)** the mean accuracy and **(B)** the mean RTs across participants while the error bars shows the 95% confidence intervals. While there was no significant difference in terms of accuracy, game RT was significantly faster than RT in the experiment (***p < 0.001).

The response accuracy between the two versions was comparable (*V* = 480, *p* = 0.53). On the other hand, RT for the gamified version was significantly faster than that for the experimental version (see Figure 11). This speed advantage for the game was expected [*t*_(40)_ = 3.61, *p* < 0.001] due to the inclusion of the coin-reward gamification feature. To look at whether the RT difference between the two versions affected the C slowing and P speeding effects, each participant's slowing effect was taken as the mean RT difference between C_200 and UN_200 and the speeding effect as the mean RT difference between UN_0 and P_0. Dependent *t*-tests indicated no significant difference in the slowing effect [*t*_(40)_ = 0.62, *p* = 0.54] or the speeding effect [t_(40)_ = −0.12, *p* = 0.91] between the two versions, in spite of the overall RT difference.

### Discussion

Experiment 4 found the expected C slowing and P speeding effects of interest. The results suggest that having the 4 conditions with −200 SOA for C and −200 SOA UN as baseline for the C effect, and 0 SOA for P with 0 SOA UN as baseline for the P effect elicits the experimental effects of interest. The gamified version produced distractor effects that were at least as strong as the corresponding experimental version in spite of the speed motivating gamification elements used in the game. These results demonstrate the validity of using a gamified version of the experiment in measuring picture-word interference and facilitation.

## General Discussion

From the four experiments, we conclude that the PWI paradigm with covert retrieval, with and without gamification, can elicit both phonological facilitation and categorical interference effects, and that the phonological facilitation is optimally induced with a later SOA (0 ms) than the categorical facilitation (−200 ms). These findings support the theories that categorical interference happens at the earlier lexical selection stage while phonological facilitation occurs at the later phonological encoding stage. Importantly, both processes are independent of the articulatory process. This result directly contradicts the response exclusion account that picture-word interference occurs at the verbal response stage, and supports the selection by competition account that semantic interference takes place at the lexical selection stage, where categorical distractors are activated as competitors against the target words, well before initiation of the articulatory motor act. Our findings also showed that, although phonological facilitation happens at the phonological retrieval stage immediately preceding verbal articulation, the facilitation effect is independent of articulation, which, to the best of our knowledge, is a finding that has not been explicitly tested previously. In consequence, our design of a PWI paradigm with button-pressing covert retrieval, with and without gamification, can be utilized in neuroimaging studies exploring the spatiotemporal activation of cortical areas during word retrieval without worrying about articulatory movement artifacts.

Moreover, the gamification results have important implications on improving online experiments. As suggested by the overall faster RT in the gamified PWI paradigm observed in our study, gamification can counter the slowed RT potentially caused by distraction, low engagement, and overall decreased control over participants found in online experiments (Semmelmann and Weigelt, 2017). The successful gamification of the covert PWI paradigm in eliciting the phonological facilitation and categorical interference effects suggests that gamification can be utilized as a more engaging online experimental method that potentially improves the boredom and inattention inherent to such testing conditions (Brehman et al., 2009), behaviorally reflected as faster RT. Furthermore, the phonological facilitation and categorical interference effects observed in the PWI paradigm and its gamification may serve as potential probes for assessing word-finding difficulties in aging, relating directly to two well-known hypotheses for explaining age-related increases in the tip-of-the-tongue (TOT) phenomenon—the Transmission Deficit Hypothesis (TDH; e.g., MacKay and Burke, 1990) and the Inhibition Deficit Hypothesis (IDH; e.g., Zacks and Hasher, 1994). The TDH proposes that TOT increases with age due to weakened connections between a word's semantic/syntactic representations and its phonological form in top-down word activation transmission (Mackay, 1988; MacKay and Burke, 1990). As a result, older adults require stronger bottom-up phonological priming to restore the word activation process compared to young adults. Once the priming is strong enough, older adults benefit from the phonological cues more than young adults (James and Burke, 2000; White and Abrams, 2002; Abrams et al., 2007; Ouyang et al., 2020). The IDH argues that the age-related increase in TOT stems from older adults' deficits in inhibiting competing words that come to mind (Hasher and Zacks, 1988; Zacks and Hasher, 1994). For example, the categorical distractors in the PWI paradigm are alternative words that fulfill the semantic/syntactic representations of the target picture to be named, forming strong competition for target word activation. Developing phonological facilitation and categorical interference effects into word-finding difficulty measures for older adults may allow us to investigate the two age-related TOT hypotheses, while enabling a word-finding assessment that is more sensitive than the standardized single-word confrontational naming test, which only measures naming accuracy. Furthermore, the gamification of the PWI-based paradigm, as validated in this study, can enhance its convenience and degree of user engagement to support its potential application as an online assessment.

## Conclusion

In four experiments, we examined the time course of phonological facilitation and categorical interference during picture naming and the potential independence of those effects from verbal articulation using a PWI paradigm with covert name retrieval. A gamified version of the paradigm was found to reliably induce the facilitation and interference effects regardless of the faster overall response speed compared to the experimental version. In both versions, we observed that categorical interference requires the distractor word to interfere at an earlier timepoint of picture naming (−200 ms SOA) while phonological facilitation is preferably elicited with the phonological distractor priming the picture name at a later timepoint (0 ms SOA). Moreover, both categorical interference and phonological facilitation effects are independent of the articulatory process in word production. These results support the selection by competition account that categorical interference is a result of competition at the more up-stream lexical selection stage (Damian et al., 2001; Belke et al., 2005; Schnur et al., 2006; Abdel Rahman and Melinger, 2011) and that phonological facilitation occurs at the more down-stream phonological encoding stage before phonetic planning for articulation (Schriefers et al., 1990; Meyer and Schriefers, 1991; Roelofs, 1992; Meyer, 1996; de Zubicaray et al., 2002; de Zubicaray and McMahon, 2009). Aside from the supports for word production theories, the covert PWI task developed in this study possesses practical advantages for neuroimaging studies and word production assessment. The covert PWI task and its gamification can be utilized in neuroimaging studies to investigate the neural mechanisms of word retrieval without articulatory artifacts. This study also support gamification as a method to improve participants engagement, reflected as faster reaction time, in online cognitive experiments. Moreover, the phonological facilitation and categorical interference effects can be used to investigate the deterioration of word production mechanisms in the aging process, and potentially enable a more engaging, convenient, and sensitive word-finding difficulty assessment that incorporates not only the measurement of word retrieval accuracy but also reaction time.

## Transparency and Openness

We report all data exclusion, all manipulation, and all measures in the study. All data, materials, codes, and Supplementary Materials, behind this analysis have been made publicly available on OSF repository named “Data, materials, and code for: Picture-word interference effects are robust with covert retrieval, with and without gamification” and can be accessed at doi: 10.17605/OSF.IO/K6NDP; https://osf.io/k6ndp/?view_only=c851b31fffe34a719f3205d3b8a91a9f. Data were analyzed using R, version 4.0.2 (R Core Team, 2020) and the visualization package ggplot2, version 3.3.2 (Wickham, 2016). This study's design and its analysis were not pre-registered.

## Data Availability Statement

The datasets presented in this study can be found in online repositories. The names of the repository/repositories and accession number(s) can be found below: Data, materials, and code for: Picture-word interference effects are robust with covert retrieval, with and without gamification at https://osf.io/k6ndp/?view_only=c851b31fffe34a719f3205d3b8a91a9f.

## Ethics Statement

The studies involving human participants were reviewed and approved by Research Ethics Boards at Baycrest Hospital and at University of Toronto. The Ethics Committee waived the requirement of written informed consent for participation.

## Author Contributions

HW and JM contributed to the conception and design of the study. MC and YH managed the gamification software development. HW designed the materials, collected the data, analyzed the data, and wrote the first draft of the manuscript. JM and MC reviewed the manuscript and supervised the project. All authors have read and approved the submitted version of the manuscript.

## Funding

This study was funded by Natural Sciences and Engineering Research Council's Discovery Grant (No. RGPIN-2019-06515) and Canada Research Chairs Program (No. CRC-2016-00140).

## Conflict of Interest

The authors declare that the research was conducted in the absence of any commercial or financial relationships that could be construed as a potential conflict of interest.

## Publisher's Note

All claims expressed in this article are solely those of the authors and do not necessarily represent those of their affiliated organizations, or those of the publisher, the editors and the reviewers. Any product that may be evaluated in this article, or claim that may be made by its manufacturer, is not guaranteed or endorsed by the publisher.
