# Supplementary material for: Picture-Word Interference Effects Are Robust With Covert Retrieval, With and Without Gamification
Source: Front Psychol. 2022 Jan 20;12:825020. doi: 10.3389/fpsyg.2021.825020 (PMC8811038; doi:10.3389/fpsyg.2021.825020)
Supplement: Supplementary file 1 [file Table_1.DOCX]

**Supplementary data**

# Picture and word lists

## EXPERIMENT 1

### List A:

|  | Target pictures | Target distractor words | Target distractor type | Non-target pictures | Non-target distractor words | Non-target distractor type |
| --- | --- | --- | --- | --- | --- | --- |
| 1 | pyramid.jpg | Egypt | A | straw.jpg | beverage | A |
| 2 | lid.jpg | pot | A | tag.jpg | price | A |
| 3 | sword.png | axe | C | chimney.jpg | fireplace | A |
| 4 | billboard.jpg | power | UN | pajama.png | underwear | C |
| 5 | clipboard.jpg | stranger | UN | grape.png | cherry | C |
| 6 | bed.jpg | sleep | A | knife.png | fork | C |
| 7 | seaweed.png | sequel | P | starfish.jpg | starter | P |
| 8 | road.jpg | sidewalk | C | octopus.jpg | oxygen | P |
| 9 | wizard.jpg | magician | C | pasta.jpg | part | P |
| 10 | mermaid.png | mercy | P | flag.jpg | rocket | UN |
| 11 | diamond.jpg | diagram | P | fish.jpg | lobby | UN |
| 12 | salad.jpg | problem | UN | lettuce.jpg | committee | UN |
| 13 | cupcake.jpg | pie | C | monkey.jpeg | banana | A |
| 14 | fork.jpg | forces | P | cave.jpg | den | A |
| 15 | duck.jpg | duchess | P | bowtie.jpeg | suit | A |
| 16 | steak.jpg | people | UN | avocado.jpg | pear | C |
| 17 | neck.png | city | UN | bench.jpg | seat | C |
| 18 | book.jpg | pamphlet | C | pizza.jpg | bread | C |
| 19 | milk.jpg | cow | A | panda.jpg | panel | P |
| 20 | chalk.jpg | choice | P | Bow.jpg | bone | P |
| 21 | shark.jpg | whale | C | jam.png | jazz | P |
| 22 | lipstick.jpg | gloss | A | worm.png | freedom | UN |
| 23 | ink.jpg | print | A | lasagna.jpg | memory | UN |
| 24 | bank.png | speed | UN | olive.jpg | sacrifice | UN |
| 25 | Stool.jpg | sugar | UN | umbrella.jpg | rain | A |
| 26 | funnel.jpg | tube | C | pillow.jpg | bed | A |
| 27 | camel.jpg | calorie | P | telescope.jpg | stars | A |
| 28 | snail.jpg | frog | A | match.jpg | flame | C |
| 29 | owl.jpg | parrot | C | flamingo.jpg | ostrich | C |
| 30 | shovel.png | frame | UN | doorknob.jpeg | lever | C |
| 31 | eagle.jpg | claws | A | saw.jpg | sausage | P |
| 32 | pill.jpg | pilgrim | P | scarf.jpg | scholar | P |
| 33 | towel.jpg | wipe | C | celery.jpg | seller | P |
| 34 | barrel.jpg | dream | UN | banana.jpg | verdict | UN |
| 35 | apple.jpg | apathy | P | Knee.jpg | dinner | UN |
| 36 | cereal.jpg | milk | A | lightning.jpg | default | UN |
| 37 | apron.jpg | cook | A | swing.jpg | playground | A |
| 38 | penguin.jpg | model | UN | beach.jpg | sand | A |
| 39 | button.jpg | coat | A | leg.jpg | pants | A |
| 40 | popcorn.jpg | chips | C | mustache.png | beard | C |
| 41 | dolphin.jpg | sea | A | crib.jpg | fence | C |
| 42 | skeleton.jpg | race | UN | volcano.jpeg | hill | C |
| 43 | iron.jpg | Ireland | P | tooth.jpg | tour | P |
| 44 | moon.jpg | planet | C | mosquito.jpg | mistake | P |
| 45 | fountain.jpg | founder | P | statue.jpg | status | P |
| 46 | Pumpkin.jpg | squash | C | dog.png | material | UN |
| 47 | oven.jpg | sheet | UN | lava.png | energy | UN |
| 48 | chain.jpg | chamber | P | arm.png | breath | UN |
| 49 | paper.jpg | ego | UN | key.jpg | gate | A |
| 50 | door.jpg | window | C | tomato.jpeg | sauce | A |
| 51 | elevator.jpeg | glance | UN | vacuum.png | carpet | A |
| 52 | razor.jpg | shaving | A | cow.jpg | buffalo | C |
| 53 | binder.jpg | bias | P | zebra.jpg | horse | C |
| 54 | hammer.jpg | nail | A | bee.jpg | fly | C |
| 55 | stapler.jpg | rabbit | UN | asparagus.jpg | ascent | P |
| 56 | chandelier.jpg | lamp | C | grass.jpg | gram | P |
| 57 | ladder.jpg | lactose | P | peach.png | peak | P |
| 58 | eraser.jpg | pencil | A | eye.png | giant | UN |
| 59 | tiger.jpg | cat | C | cookie.jpeg | trait | UN |
| 60 | Anchor.jpg | angle | P | teabag.jpg | law | UN |
| 61 | helmet.jpg | health | P | kangaroo.jpg | pouch | A |
| 62 | bat.jpg | cave | A | camera.jpg | picture | A |
| 63 | boat.jpeg | bonus | P | radio.jpeg | music | A |
| 64 | robot.jpg | machine | C | bus.png | car | C |
| 65 | wallet.jpg | purse | C | igloo.jpg | shack | C |
| 66 | tent.jpg | canvas | C | turkey.jpg | chicken | C |
| 67 | elephant.jpg | hardship | UN | farm.jpg | father | P |
| 68 | bullet.jpg | butchery | P | hourglass.jpg | ourselves | P |
| 69 | shirt.jpg | pin | UN | Wolf.jpg | woman | P |
| 70 | nest.jpeg | bird | A | gorilla.jpg | inch | UN |
| 71 | flashlight.jpg | thinker | UN | glove.jpg | idea | UN |
| 72 | cigarette.jpg | lungs | A | broccoli.jpg | excitement | UN |

### List B:

|  | Target pictures | Target distractor words | Target distractor type | Non-target pictures | Non-target distractor words | Non-target distractor type |
| --- | --- | --- | --- | --- | --- | --- |
| 1 | brick.jpg | wall | A | Sushi.jpg | Japan | A |
| 2 | clock.jpg | closet | P | cage.jpeg | bird | A |
| 3 | desk.jpg | table | C | Ladybug.jpg | labor | P |
| 4 | snake.jpg | lizard | C | stamp.jpg | blood | UN |
| 5 | garlic.jpg | garden | P | fly.jpg | beetle | C |
| 6 | smoke.jpg | burning | A | wing.png | winner | P |
| 7 | mask.jpeg | veil | C | bamboo.jpg | panda | A |
| 8 | peacock.jpg | peace | P | shrimp.png | lobster | C |
| 9 | skunk.jpg | orange | UN | microscope.jpg | lung | UN |
| 10 | tank.jpg | war | A | mango.jpg | glimpse | UN |
| 11 | truck.jpg | poetry | UN | microwave.jpg | mile | P |
| 12 | lock.jpg | spirit | UN | battery.jpg | charger | C |
| 13 | bagel.png | baseline | P | frisbee.png | disc | C |
| 14 | Bull.jpg | voyage | UN | Jellyfish.jpg | squid | C |
| 15 | candle.jpg | torch | C | shelf.jpg | book | A |
| 16 | well.jpg | seminar | UN | Sheep.jpg | quality | UN |
| 17 | medal.jpg | ribbon | C | butterfly.jpg | twin | UN |
| 18 | pencil.jpg | notebook | A | crutch.jpg | crust | P |
| 19 | pool.jpg | pooch | P | mop.jpg | broom | C |
| 20 | shell.jpg | beach | A | plug.jpg | outlet | A |
| 21 | squirrel.jpg | chipmunk | C | earring.png | outcome | UN |
| 22 | tail.jpeg | stadium | UN | tray.jpeg | tracer | P |
| 23 | tunnel.jpg | touch | P | ashtray.png | cigarette | A |
| 24 | windmill.jpg | energy | A | sandwich.jpg | sanity | P |
| 25 | brain.jpg | pillow | UN | egg.jpg | card | UN |
| 26 | cannon.jpg | candidate | P | soap.jpg | shampoo | C |
| 27 | bacon.jpg | ham | C | crab.jpg | craft | P |
| 28 | clown.jpg | cloud | P | blueberry.jpg | college | UN |
| 29 | corn.png | field | A | coffee.jpg | tea | C |
| 30 | fan.jpg | blower | C | mousetrap.jpg | bill | UN |
| 31 | horn.png | beep | A | sponge.jpg | dishes | A |
| 32 | lemon.jpg | legend | P | mummy.png | coffin | A |
| 33 | Onion.jpg | curve | UN | drum.png | drug | P |
| 34 | acorn.jpg | walnut | C | mushroom.jpg | fungus | C |
| 35 | spoon.jpg | orthodox | UN | cup.jpg | custom | P |
| 36 | violin.jpg | orchestra | A | window.jpg | glass | A |
| 37 | beaver.jpg | squirrel | C | koala.jpg | wombat | C |
| 38 | Blender.jpg | blemish | P | tree.jpg | forest | A |
| 39 | cucumber.jpg | melon | C | eyelash.jpg | mascara | A |
| 40 | ear.jpeg | sound | A | jeep.jpg | genius | P |
| 41 | feather.jpg | fellowship | P | king.jpg | queen | C |
| 42 | escalator.jpg | magnet | UN | strawberry.jpg | meat | UN |
| 43 | hanger.jpg | coat | A | dragonfly.png | clothes | UN |
| 44 | lighter.png | lion | P | trophy.jpg | prize | C |
| 45 | spider.jpg | scorpion | C | broom.jpg | dust | A |
| 46 | projector.jpg | missile | UN | horseshoe.png | nature | UN |
| 47 | ruler.jpg | length | A | pipe.jpg | pint | P |
| 48 | toaster.jpg | intention | UN | tornado.jpg | torrent | P |
| 49 | cactus.jpg | desert | A | pump.jpg | pub | P |
| 50 | compass.jpg | company | P | roof.jpg | cover | C |
| 51 | cross.jpg | church | A | radish.jpg | racket | P |
| 52 | briefcase.png | bag | C | airbag.jpg | car | A |
| 53 | glass.jpg | math | UN | Kiwi.jpg | keynote | P |
| 54 | horse.jpg | hormone | P | ham.jpg | lawyer | UN |
| 55 | Lighthouse.jpg | tower | C | bridge.jpg | link | C |
| 56 | mattress.jpg | bed | A | tape.jpg | bandage | A |
| 57 | Moose.jpg | mover | P | frog.jpg | toad | C |
| 58 | nose.jpg | film | UN | cherry.jpg | estate | UN |
| 59 | rice.jpg | oat | C | light bulb.jpg | electricity | A |
| 60 | fence.png | tab | UN | screw.jpg | action | UN |
| 61 | belt.jpg | pants | A | taco.jpg | march | UN |
| 62 | bucket.jpg | can | C | map.jpeg | direction | A |
| 63 | carrot.jpg | celery | C | ramp.jpeg | slope | C |
| 64 | doughnut.jpeg | donor | P | piano.jpg | peeler | P |
| 65 | eggplant.jpg | summer | UN | glue.png | police | UN |
| 66 | goat.jpg | ghost | P | rainbow.jpg | color | A |
| 67 | kite.jpg | glider | C | candy.jpg | candor | P |
| 68 | plate.jpg | food | A | potato.jpg | chips | A |
| 69 | ant.jpg | anvil | P | rope.jpg | thread | C |
| 70 | spotlight.jpg | fame | A | moth.png | wasp | C |
| 71 | Toilet.jpg | scene | UN | Ostrich.jpg | soup | UN |
| 72 | wheat.jpg | location | UN | elbow.jpg | elf | P |

## EXPERIMENT 2 & 3

### List A

|  | Target pictures | Target distractor words | Target distractor type | Non-target pictures | Non-target distractor words | Non-target distractor type |
| --- | --- | --- | --- | --- | --- | --- |
| 1 | pyramid.jpg |  | No | straw.jpg |  | No |
| 2 | lid.jpg |  | No | tag.jpg |  | No |
| 3 | sword.png | axe | C | chimney.jpg |  | No |
| 4 | billboard.jpg | power | UN | pajama.png | underwear | C |
| 5 | clipboard.jpg | stranger | UN | grape.png | cherry | C |
| 6 | bed.jpg |  | No | knife.png | fork | C |
| 7 | seaweed.png | sequel | P | starfish.jpg | starter | P |
| 8 | road.jpg | sidewalk | C | octopus.jpg | oxygen | P |
| 9 | wizard.jpg | magician | C | pasta.jpg | part | P |
| 10 | mermaid.png | mercy | P | flag.jpg | rocket | UN |
| 11 | diamond.jpg | diagram | P | fish.jpg | lobby | UN |
| 12 | salad.jpg | problem | UN | lettuce.jpg | committee | UN |
| 13 | cupcake.jpg | pie | C | monkey.jpeg |  | No |
| 14 | fork.jpg | forces | P | cave.jpg |  | No |
| 15 | duck.jpg | duchess | P | bowtie.jpeg |  | No |
| 16 | steak.jpg | people | UN | avocado.jpg | pear | C |
| 17 | neck.png | city | UN | bench.jpg | seat | C |
| 18 | book.jpg | pamphlet | C | pizza.jpg | bread | C |
| 19 | milk.jpg |  | No | panda.jpg | panel | P |
| 20 | chalk.jpg | choice | P | Bow.jpg | bone | P |
| 21 | shark.jpg | whale | C | jam.png | jazz | P |
| 22 | lipstick.jpg |  | No | worm.png | freedom | UN |
| 23 | ink.jpg |  | No | lasagna.jpg | memory | UN |
| 24 | bank.png | speed | UN | olive.jpg | sacrifice | UN |
| 25 | Stool.jpg | sugar | UN | umbrella.jpg |  | No |
| 26 | funnel.jpg | tube | C | pillow.jpg |  | No |
| 27 | camel.jpg | calorie | P | telescope.jpg |  | No |
| 28 | snail.jpg |  | No | match.jpg | flame | C |
| 29 | owl.jpg | parrot | C | flamingo.jpg | ostrich | C |
| 30 | shovel.png | frame | UN | doorknob.jpeg | lever | C |
| 31 | eagle.jpg |  | No | saw.jpg | sausage | P |
| 32 | pill.jpg | pilgrim | P | scarf.jpg | scholar | P |
| 33 | towel.jpg | wipe | C | celery.jpg | seller | P |
| 34 | barrel.jpg | dream | UN | banana.jpg | verdict | UN |
| 35 | apple.jpg | apathy | P | Knee.jpg | dinner | UN |
| 36 | cereal.jpg |  | No | lightning.jpg | default | UN |
| 37 | apron.jpg |  | No | swing.jpg |  | No |
| 38 | penguin.jpg | model | UN | beach.jpg |  | No |
| 39 | button.jpg |  | No | leg.jpg |  | No |
| 40 | popcorn.jpg | chips | C | mustache.png | beard | C |
| 41 | dolphin.jpg |  | No | crib.jpg | fence | C |
| 42 | skeleton.jpg | race | UN | volcano.jpeg | hill | C |
| 43 | iron.jpg | Ireland | P | tooth.jpg | tour | P |
| 44 | moon.jpg | planet | C | mosquito.jpg | mistake | P |
| 45 | fountain.jpg | founder | P | statue.jpg | status | P |
| 46 | Pumpkin.jpg | squash | C | dog.png | material | UN |
| 47 | oven.jpg | sheet | UN | lava.png | energy | UN |
| 48 | chain.jpg | chamber | P | arm.png | breath | UN |
| 49 | paper.jpg | ego | UN | key.jpg |  | No |
| 50 | door.jpg | window | C | tomato.jpeg |  | No |
| 51 | elevator.jpeg | glance | UN | vacuum.png |  | No |
| 52 | razor.jpg |  | No | cow.jpg | buffalo | C |
| 53 | binder.jpg | bias | P | zebra.jpg | horse | C |
| 54 | hammer.jpg |  | No | bee.jpg | fly | C |
| 55 | stapler.jpg | rabbit | UN | asparagus.jpg | ascent | P |
| 56 | chandelier.jpg | lamp | C | grass.jpg | gram | P |
| 57 | ladder.jpg | lactose | P | peach.png | peak | P |
| 58 | eraser.jpg |  | No | eye.png | giant | UN |
| 59 | tiger.jpg | cat | C | cookie.jpeg | trait | UN |
| 60 | Anchor.jpg | angle | P | teabag.jpg | law | UN |
| 61 | helmet.jpg | health | P | kangaroo.jpg |  | No |
| 62 | bat.jpg |  | No | camera.jpg |  | No |
| 63 | boat.jpeg | bonus | P | radio.jpeg |  | No |
| 64 | robot.jpg | machine | C | bus.png | car | C |
| 65 | wallet.jpg | purse | C | igloo.jpg | shack | C |
| 66 | tent.jpg | canvas | C | turkey.jpg | chicken | C |
| 67 | elephant.jpg | hardship | UN | farm.jpg | father | P |
| 68 | bullet.jpg | butchery | P | hourglass.jpg | ourselves | P |
| 69 | shirt.jpg | pin | UN | Wolf.jpg | woman | P |
| 70 | nest.jpeg |  | No | gorilla.jpg | inch | UN |
| 71 | flashlight.jpg | thinker | UN | glove.jpg | idea | UN |
| 72 | cigarette.jpg |  | No | broccoli.jpg | excitement | UN |

### List B

|  | Target pictures | Target distractor words | Target distractor type | Non-target pictures | Non-target distractor words | Non-target distractor type |
| --- | --- | --- | --- | --- | --- | --- |
| 1 | brick.jpg |  | No | Sushi.jpg |  | No |
| 2 | clock.jpg | closet | P | cage.jpeg |  | No |
| 3 | desk.jpg | table | C | Ladybug.jpg | labor | P |
| 4 | snake.jpg | lizard | C | stamp.jpg | blood | UN |
| 5 | garlic.jpg | garden | P | fly.jpg | beetle | C |
| 6 | smoke.jpg |  | No | wing.png | winner | P |
| 7 | mask.jpeg | veil | C | bamboo.jpg |  | No |
| 8 | peacock.jpg | peace | P | shrimp.png | lobster | C |
| 9 | skunk.jpg | orange | UN | microscope.jpg | lung | UN |
| 10 | tank.jpg |  | No | mango.jpg | glimpse | UN |
| 11 | truck.jpg | poetry | UN | microwave.jpg | mile | P |
| 12 | lock.jpg | spirit | UN | battery.jpg | charger | C |
| 13 | bagel.png | baseline | P | frisbee.png | disc | C |
| 14 | Bull.jpg | voyage | UN | Jellyfish.jpg | squid | C |
| 15 | candle.jpg | torch | C | shelf.jpg |  | No |
| 16 | well.jpg | seminar | UN | Sheep.jpg | quality | UN |
| 17 | medal.jpg | ribbon | C | butterfly.jpg | twin | UN |
| 18 | pencil.jpg |  | No | crutch.jpg | crust | P |
| 19 | pool.jpg | pooch | P | mop.jpg | broom | C |
| 20 | shell.jpg |  | No | plug.jpg |  | No |
| 21 | squirrel.jpg | chipmunk | C | earring.png | outcome | UN |
| 22 | tail.jpeg | stadium | UN | tray.jpeg | tracer | P |
| 23 | tunnel.jpg | touch | P | ashtray.png |  | No |
| 24 | windmill.jpg |  | No | sandwich.jpg | sanity | P |
| 25 | brain.jpg | pillow | UN | egg.jpg | card | UN |
| 26 | cannon.jpg | candidate | P | soap.jpg | shampoo | C |
| 27 | bacon.jpg | ham | C | crab.jpg | craft | P |
| 28 | clown.jpg | cloud | P | blueberry.jpg | college | UN |
| 29 | corn.png |  | No | coffee.jpg | tea | C |
| 30 | fan.jpg | blower | C | mousetrap.jpg | bill | UN |
| 31 | horn.png |  | No | sponge.jpg |  | No |
| 32 | lemon.jpg | legend | P | mummy.png |  | No |
| 33 | Onion.jpg | curve | UN | drum.png | drug | P |
| 34 | acorn.jpg | walnut | C | mushroom.jpg | fungus | C |
| 35 | spoon.jpg | orthodox | UN | cup.jpg | custom | P |
| 36 | violin.jpg |  | No | window.jpg |  | No |
| 37 | beaver.jpg | squirrel | C | koala.jpg | wombat | C |
| 38 | Blender.jpg | blemish | P | tree.jpg |  | No |
| 39 | cucumber.jpg | melon | C | eyelash.jpg |  | No |
| 40 | ear.jpeg |  | No | jeep.jpg | genius | P |
| 41 | feather.jpg | fellowship | P | king.jpg | queen | C |
| 42 | escalator.jpg | magnet | UN | strawberry.jpg | meat | UN |
| 43 | hanger.jpg |  | No | dragonfly.png | clothes | UN |
| 44 | lighter.png | lion | P | trophy.jpg | prize | C |
| 45 | spider.jpg | scorpion | C | broom.jpg |  | No |
| 46 | projector.jpg | missile | UN | horseshoe.png | nature | UN |
| 47 | ruler.jpg |  | No | pipe.jpg | pint | P |
| 48 | toaster.jpg | intention | UN | tornado.jpg | torrent | P |
| 49 | cactus.jpg |  | No | pump.jpg | pub | P |
| 50 | compass.jpg | company | P | roof.jpg | cover | C |
| 51 | cross.jpg |  | No | radish.jpg | racket | P |
| 52 | briefcase.png | bag | C | airbag.jpg |  | No |
| 53 | glass.jpg | math | UN | Kiwi.jpg | keynote | P |
| 54 | horse.jpg | hormone | P | ham.jpg | lawyer | UN |
| 55 | Lighthouse.jpg | tower | C | bridge.jpg | link | C |
| 56 | mattress.jpg |  | No | tape.jpg |  | No |
| 57 | Moose.jpg | mover | P | frog.jpg | toad | C |
| 58 | nose.jpg | film | UN | cherry.jpg | estate | UN |
| 59 | rice.jpg | oat | C | light bulb.jpg |  | No |
| 60 | fence.png | tab | UN | screw.jpg | action | UN |
| 61 | belt.jpg |  | No | taco.jpg | march | UN |
| 62 | bucket.jpg | can | C | map.jpeg |  | No |
| 63 | carrot.jpg | celery | C | ramp.jpeg | slope | C |
| 64 | doughnut.jpeg | donor | P | piano.jpg | peeler | P |
| 65 | eggplant.jpg | summer | UN | glue.png | police | UN |
| 66 | goat.jpg | ghost | P | rainbow.jpg |  | No |
| 67 | kite.jpg | glider | C | candy.jpg | candor | P |
| 68 | plate.jpg |  | No | potato.jpg |  | No |
| 69 | ant.jpg | anvil | P | rope.jpg | thread | C |
| 70 | spotlight.jpg |  | No | moth.png | wasp | C |
| 71 | Toilet.jpg | scene | UN | Ostrich.jpg | soup | UN |
| 72 | wheat.jpg | location | UN | elbow.jpg | elf | P |

## EXPERIMENT 4

### List A

|  | Target pictures | Target distractor words | Target distractor type | Non-target pictures | Non-target distractor words | Non-target distractor type |
| --- | --- | --- | --- | --- | --- | --- |
| 1 | pyramid.jpg | humor | UN | straw.jpg | yacht | UN |
| 2 | lid.jpg | failure | UN | tag.jpg | river | UN |
| 3 | sword.png | axe | C | chimney.jpg | crisis | UN |
| 4 | billboard.jpg | power | UN | pajama.png | underwear | C |
| 5 | clipboard.jpg | stranger | UN | grape.png | cherry | C |
| 6 | bed.jpg | relief | UN | knife.png | fork | C |
| 7 | seaweed.png | sequel | P | starfish.jpg | starter | P |
| 8 | road.jpg | sidewalk | C | octopus.jpg | oxygen | P |
| 9 | wizard.jpg | magician | C | pasta.jpg | part | P |
| 10 | mermaid.png | mercy | P | flag.jpg | rocket | UN |
| 11 | diamond.jpg | diagram | P | fish.jpg | lobby | UN |
| 12 | salad.jpg | problem | UN | lettuce.jpg | committee | UN |
| 13 | cupcake.jpg | pie | C | monkey.jpeg | seed | UN |
| 14 | fork.jpg | forces | P | cave.jpg | tax | UN |
| 15 | duck.jpg | duchess | P | bowtie.jpeg | tear | UN |
| 16 | steak.jpg | people | UN | avocado.jpg | pear | C |
| 17 | neck.png | city | UN | bench.jpg | seat | C |
| 18 | book.jpg | pamphlet | C | pizza.jpg | bread | C |
| 19 | milk.jpg | budget | UN | panda.jpg | panel | P |
| 20 | chalk.jpg | choice | P | Bow.jpg | bone | P |
| 21 | shark.jpg | whale | C | jam.png | jazz | P |
| 22 | lipstick.jpg | prison | UN | worm.png | freedom | UN |
| 23 | ink.jpg | needle | UN | lasagna.jpg | memory | UN |
| 24 | bank.png | speed | UN | olive.jpg | sacrifice | UN |
| 25 | Stool.jpg | sugar | UN | umbrella.jpg | creek | UN |
| 26 | funnel.jpg | tube | C | pillow.jpg | beauty | UN |
| 27 | camel.jpg | calorie | P | telescope.jpg | merit | UN |
| 28 | snail.jpg | chime | UN | match.jpg | flame | C |
| 29 | owl.jpg | parrot | C | flamingo.jpg | ostrich | C |
| 30 | shovel.png | frame | UN | doorknob.jpeg | lever | C |
| 31 | eagle.jpg | core | UN | saw.jpg | sausage | P |
| 32 | pill.jpg | pilgrim | P | scarf.jpg | scholar | P |
| 33 | towel.jpg | wipe | C | celery.jpg | seller | P |
| 34 | barrel.jpg | dream | UN | banana.jpg | verdict | UN |
| 35 | apple.jpg | apathy | P | Knee.jpg | dinner | UN |
| 36 | cereal.jpg | answer | UN | lightning.jpg | default | UN |
| 37 | apron.jpg | layer | UN | swing.jpg | frost | UN |
| 38 | penguin.jpg | model | UN | beach.jpg | wage | UN |
| 39 | button.jpg | oval | UN | leg.jpg | gap | UN |
| 40 | popcorn.jpg | chips | C | mustache.png | beard | C |
| 41 | dolphin.jpg | sale | UN | crib.jpg | fence | C |
| 42 | skeleton.jpg | race | UN | volcano.jpeg | hill | C |
| 43 | iron.jpg | Ireland | P | tooth.jpg | tour | P |
| 44 | moon.jpg | planet | C | mosquito.jpg | mistake | P |
| 45 | fountain.jpg | founder | P | statue.jpg | status | P |
| 46 | Pumpkin.jpg | squash | C | dog.png | material | UN |
| 47 | oven.jpg | sheet | UN | lava.png | energy | UN |
| 48 | chain.jpg | chamber | P | arm.png | breath | UN |
| 49 | paper.jpg | ego | UN | key.jpg | blog | UN |
| 50 | door.jpg | window | C | tomato.jpeg | cousin | UN |
| 51 | elevator.jpeg | glance | UN | vacuum.png | school | UN |
| 52 | razor.jpg | balance | UN | cow.jpg | buffalo | C |
| 53 | binder.jpg | bias | P | zebra.jpg | horse | C |
| 54 | hammer.jpg | logic | UN | bee.jpg | fly | C |
| 55 | stapler.jpg | rabbit | UN | asparagus.jpg | ascent | P |
| 56 | chandelier.jpg | lamp | C | grass.jpg | gram | P |
| 57 | ladder.jpg | lactose | P | peach.png | peak | P |
| 58 | eraser.jpg | den | UN | eye.png | giant | UN |
| 59 | tiger.jpg | cat | C | cookie.jpeg | trait | UN |
| 60 | Anchor.jpg | angle | P | teabag.jpg | law | UN |
| 61 | helmet.jpg | health | P | kangaroo.jpg | average | UN |
| 62 | bat.jpg | campus | UN | camera.jpg | reason | UN |
| 63 | boat.jpeg | bonus | P | radio.jpeg | growth | UN |
| 64 | robot.jpg | machine | C | bus.png | car | C |
| 65 | wallet.jpg | purse | C | igloo.jpg | shack | C |
| 66 | tent.jpg | canvas | C | turkey.jpg | chicken | C |
| 67 | elephant.jpg | hardship | UN | farm.jpg | father | P |
| 68 | bullet.jpg | butchery | P | hourglass.jpg | ourselves | P |
| 69 | shirt.jpg | pin | UN | Wolf.jpg | woman | P |
| 70 | nest.jpeg | center | UN | gorilla.jpg | inch | UN |
| 71 | flashlight.jpg | thinker | UN | glove.jpg | idea | UN |
| 72 | cigarette.jpg | hair | UN | broccoli.jpg | excitement | UN |

### List B

|  | Target pictures | Target distractor words | Target distractor type | Non-target pictures | Non-target distractor words | Non-target distractor type |
| --- | --- | --- | --- | --- | --- | --- |
| 1 | brick.jpg | promise | UN | Sushi.jpg | dirt | UN |
| 2 | clock.jpg | closet | P | cage.jpeg | phrase | UN |
| 3 | desk.jpg | table | C | Ladybug.jpg | labor | P |
| 4 | snake.jpg | lizard | C | stamp.jpg | blood | UN |
| 5 | garlic.jpg | garden | P | fly.jpg | beetle | C |
| 6 | smoke.jpg | belly | UN | wing.png | winner | P |
| 7 | mask.jpeg | veil | C | bamboo.jpg | survey | UN |
| 8 | peacock.jpg | peace | P | shrimp.png | lobster | C |
| 9 | skunk.jpg | orange | UN | microscope.jpg | lung | UN |
| 10 | tank.jpg | gown | UN | mango.jpg | glimpse | UN |
| 11 | truck.jpg | poetry | UN | microwave.jpg | mile | P |
| 12 | lock.jpg | spirit | UN | battery.jpg | charger | C |
| 13 | bagel.png | baseline | P | frisbee.png | disc | C |
| 14 | Bull.jpg | voyage | UN | Jellyfish.jpg | squid | C |
| 15 | candle.jpg | torch | C | shelf.jpg | palace | UN |
| 16 | well.jpg | seminar | UN | Sheep.jpg | quality | UN |
| 17 | medal.jpg | ribbon | C | butterfly.jpg | twin | UN |
| 18 | pencil.jpg | caution | UN | crutch.jpg | crust | P |
| 19 | pool.jpg | pooch | P | mop.jpg | broom | C |
| 20 | shell.jpg | airport | UN | plug.jpg | oil | UN |
| 21 | squirrel.jpg | chipmunk | C | earring.png | outcome | UN |
| 22 | tail.jpeg | stadium | UN | tray.jpeg | tracer | P |
| 23 | tunnel.jpg | touch | P | ashtray.png | saint | UN |
| 24 | windmill.jpg | hive | UN | sandwich.jpg | sanity | P |
| 25 | brain.jpg | pillow | UN | egg.jpg | card | UN |
| 26 | cannon.jpg | candidate | P | soap.jpg | shampoo | C |
| 27 | bacon.jpg | ham | C | crab.jpg | craft | P |
| 28 | clown.jpg | cloud | P | blueberry.jpg | college | UN |
| 29 | corn.png | fallacy | UN | coffee.jpg | tea | C |
| 30 | fan.jpg | blower | C | mousetrap.jpg | bill | UN |
| 31 | horn.png | savior | UN | sponge.jpg | costume | UN |
| 32 | lemon.jpg | legend | P | mummy.png | skill | UN |
| 33 | Onion.jpg | curve | UN | drum.png | drug | P |
| 34 | acorn.jpg | walnut | C | mushroom.jpg | fungus | C |
| 35 | spoon.jpg | orthodox | UN | cup.jpg | custom | P |
| 36 | violin.jpg | site | UN | window.jpg | flood | UN |
| 37 | beaver.jpg | squirrel | C | koala.jpg | wombat | C |
| 38 | Blender.jpg | blemish | P | tree.jpg | opera | UN |
| 39 | cucumber.jpg | melon | C | eyelash.jpg | influence | UN |
| 40 | ear.jpeg | host | UN | jeep.jpg | genius | P |
| 41 | feather.jpg | fellowship | P | king.jpg | queen | C |
| 42 | escalator.jpg | magnet | UN | strawberry.jpg | meat | UN |
| 43 | hanger.jpg | refuge | UN | dragonfly.png | clothes | UN |
| 44 | lighter.png | lion | P | trophy.jpg | prize | C |
| 45 | spider.jpg | scorpion | C | broom.jpg | zombie | UN |
| 46 | projector.jpg | missile | UN | horseshoe.png | nature | UN |
| 47 | ruler.jpg | fatigue | UN | pipe.jpg | pint | P |
| 48 | toaster.jpg | intention | UN | tornado.jpg | torrent | P |
| 49 | cactus.jpg | area | UN | pump.jpg | pub | P |
| 50 | compass.jpg | company | P | roof.jpg | cover | C |
| 51 | cross.jpg | award | UN | radish.jpg | racket | P |
| 52 | briefcase.png | bag | C | airbag.jpg | purple | UN |
| 53 | glass.jpg | math | UN | Kiwi.jpg | keynote | P |
| 54 | horse.jpg | hormone | P | ham.jpg | lawyer | UN |
| 55 | Lighthouse.jpg | tower | C | bridge.jpg | link | C |
| 56 | mattress.jpg | shop | UN | tape.jpg | chord | UN |
| 57 | Moose.jpg | mover | P | frog.jpg | toad | C |
| 58 | nose.jpg | film | UN | cherry.jpg | estate | UN |
| 59 | rice.jpg | oat | C | light bulb.jpg | fort | UN |
| 60 | fence.png | tab | UN | screw.jpg | action | UN |
| 61 | belt.jpg | stream | UN | taco.jpg | march | UN |
| 62 | bucket.jpg | can | C | map.jpeg | fax | UN |
| 63 | carrot.jpg | celery | C | ramp.jpeg | slope | C |
| 64 | doughnut.jpeg | donor | P | piano.jpg | peeler | P |
| 65 | eggplant.jpg | summer | UN | glue.png | police | UN |
| 66 | goat.jpg | ghost | P | rainbow.jpg | exam | UN |
| 67 | kite.jpg | glider | C | candy.jpg | candor | P |
| 68 | plate.jpg | charity | UN | potato.jpg | line | UN |
| 69 | ant.jpg | anvil | P | rope.jpg | thread | C |
| 70 | spotlight.jpg | prayer | UN | moth.png | wasp | C |
| 71 | Toilet.jpg | scene | UN | Ostrich.jpg | soup | UN |
| 72 | wheat.jpg | location | UN | elbow.jpg | elf | P |
